# Role of d-serine in intestinal ROS accumulation after sleep deprivation

**DOI:** 10.1126/sciadv.adr8592

**Published:** 2025-07-18

**Authors:** Feng Zheng, Shuai Liu, Tian Wei, Lei Wang, Yuanyuan Chang, Hao Qu, Lei Zheng

**Affiliations:** ^1^ School of Food and Biological Engineering, Hefei University of Technology, Hefei, China.; ^2^ Department of Toxicology, School of Public Health, Anhui Medical University, Hefei, China.; ^3^ Research Laboratory of Agricultural Environment and Food Safety, Anhui Modern Agricultural Industry Technology System, Hefei 230009, China.

## Abstract

Prolonged sleep deprivation (SD) results in increased accumulation of reactive oxygen species (ROS) in gut, although the underlying mechanisms remain to be elucidated. This study identifies d-serine as a crucial regulator of gut ROS during SD. Knockdown of serine racemase (SR), the enzyme responsible for d-serine production, prevents the enhanced ROS buildup during SD in *Drosophila*. Gut enterocytes (ECs) respond to γ-aminobutyric acid (GABA) signaling by producing d-serine, which influences downstream *N*-methyl-d-aspartate receptor (NMDAR) activity and modulates sleep pressure. However, the continuous demand for sleep disrupts this feedback loop. Prolonged SD leads to increased levels of d-serine in the gut, an elevated pyruvate pool in ECs, enhanced mitochondrial oxidative phosphorylation, impaired lipid metabolism in peroxisomes, and the accumulation of harmful ROS. In conclusion, our findings illuminate the metabolic alterations and brain-gut communication pathways that may contribute to the increase in gut d-serine and subsequent ROS accumulation induced by SD.

## Teaser

The role of d-serine in ROS accumulation in the gut from sleep deprivation and its links to gut-brain pathways are uncovered.

## INTRODUCTION

Sleep is a universal biological activity shared across organisms, characterized by distinct properties such as constancy, insensitivity to external stimuli, and the ability to compensate for sleep loss (*1*–*3*). The regulation of normal sleep is influenced by both the internal circadian clock and sleep homeostasis systems, which also receive input from the external environment. The circadian clock governs the rhythm of sleep throughout the day, while sleep homeostasis systems adjust sleep requirements based on variations in sleep duration and quality (*3*).

High-quality and sufficient sleep is essential for maintaining optimal physiological fitness, enabling organisms to integrate daily experiences (*2*, *4*–*6*), enabling memory consolidation, and removing waste products exchanged across the blood-brain barrier (*7*–*9*). Furthermore, adequate sleep is critical for the normal functioning of the immune system (*10*, *11*). Deficient or disrupted sleep can lead to adverse effects, including psychological disorders, metabolic issues, cognitive decline, and even mortality (*10*, *12*–*17*).

A variety of molecular mechanisms regulate numerous physiological functions and mental states influenced by sleep (*6*, *7*, *18*–*23*). Sleep deprivation (SD) specifically modulates mood alterations via dopamine activity and enhances pain perception through glutamatergic-norepinephrine pathways (*18*, *19*). Recent studies have shown that chronic SD reduces the life span of *Drosophila* due to the accumulation of a lethal dose of reactive oxygen species (ROS) in the gut (*16*). Moreover, SD has been associated with a cytokine storm–like syndrome in mice, triggered by the release of prostaglandin D2 from the central nervous system to the peripheral immune system (*12*).

The precise mechanisms linking gut ROS accumulation to premature mortality during SD remain unresolved (*12*, *16*). While independent studies using various model organisms, including zebrafish, *Drosophila*, and mice, have consistently demonstrated that SD induces gut ROS accumulation (*12*, *16*, *24*), the precise role of the gut in the context of SD is not yet fully understood, and the specific alterations in the gut that contribute to ROS accumulation during SD are still to be determined. The critical role of neurotransmitters and hormones in sleep homeostasis underscores the importance of understanding how signals between the brain and gut are regulated (*20*, *25*–*28*). These interactions likely influence the gut microbiota or signaling molecules required for ROS production during SD. Investigating these mechanisms could offer valuable insights into mitigating premature death associated with SD.

Given the specificity of the gut phenotype, we hypothesize that gut signaling molecules influencing sleep are likely involved. A recent study has shown that d-serine (d-Ser), an amino acid expressed in both the gut and the brain, promotes daily sleep but is primarily sourced from the gut (*29*). d-Ser is an endogenous amino acid that can co-activate the NMDAR1. It is synthesized from l-serine (l-Ser) by serine racemase (SR) and eliminated by d-amino acid oxidase (DAAO), which is encoded by *CG11236* and *CG12338* or eliminated by SR itself.

The abundance of d-Ser in neural and endocrine tissues linked to central nervous system signaling and glucose metabolism (*30*–*33*) suggests that its dysregulation may contribute to various diseases. For instance, reduced d-Ser levels in the brains of aged animals correlate with cognitive deficits (*34*), and the SR-mediated dehydration of d-Ser and l-Ser has been implicated in colorectal cancer progression (*34*, *35*). Thus, maintaining balanced d-Ser levels is vital for organismal health.

In this study, we investigated the influence of d-Ser on intestinal ROS accumulation and life span during SD using *Drosophila*. Our findings revealed that the *SR* knockout mutant *Drosophila* did not accumulate ROS in the gut when subjected to SD through thermogenetic methods or mechanical vibration, and they exhibited a prolonged life span. In contrast, the *DAAO* knockout mutant showed increased generation and accumulation of ROS, leading to an earlier mortality. A potential feedback loop between dorsal fan-shaped body (dFB) neurons and enterocytes (ECs) through SR–*N*-methyl-d-aspartate receptor (NMDAR1) signaling provides a mechanistic explanation for how sleep need influences gut function. Furthermore, elevated levels of d-Ser during SD were found to affect mitochondrial respiration and fatty acid metabolism, contributing to increased ROS accumulation in the gut. Thus, we propose that our results indicate d-Ser may serve as a unique indicator of the gut’s metabolic state in response to sleep demands.

## RESULTS

### Gut ROS from SD is prevented in *SRko* but worsened in *DAAOko*

**Fig. 1.**
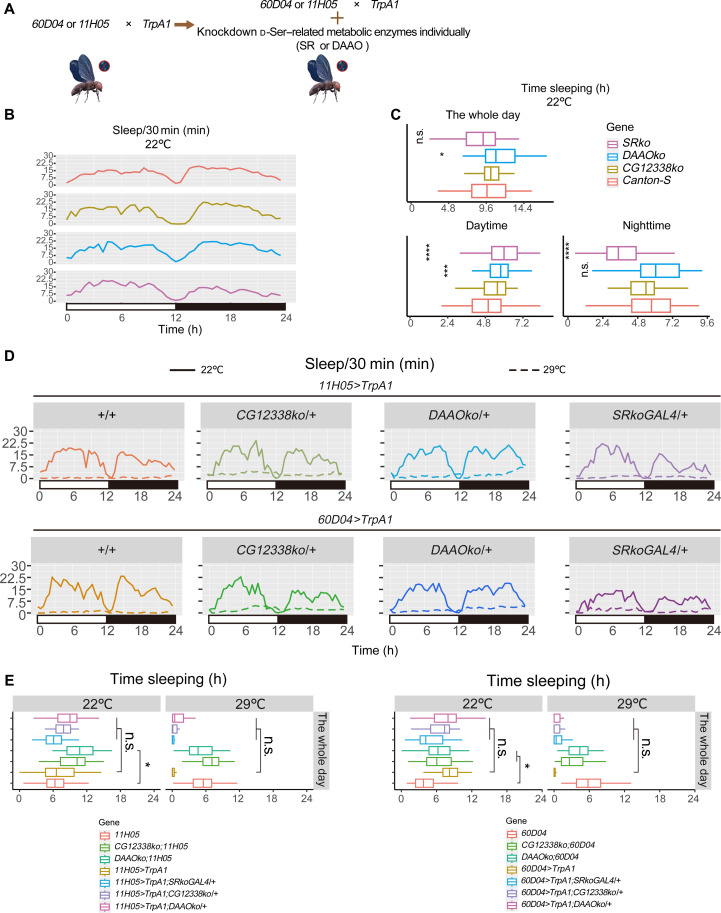
The fruit flies with knockout of *SR* or *DAAO* exhibit similar levels of SD at 29°C. (**A**) Schematic representation of the genetic manipulation of the SD model. (**B**) Sleep status of *Canton-S*, *SRko*, *DAAOko*, and *CG12338ko* at 22°C, with 2 days of sleep data consolidated. (**C**) Boxplot statistics for the sleep amounts shown in (B). *P < 0.05; ***P < 0.001; ****P < 0.0001. (**D**) Sleep status of *UAS-TrpA1/+;11H05GAL4/+*, *UAS-TrpA1/+;60D04GAL4/+*, *UAS-TrpA1/ CG12338ko;11H05GAL4/+*, *UAS-TrpA1/CG12338ko;60D04GAL4/+*, *UAS-TrpA1/DAAOko;11H05GAL4/+*, *UAS-TrpA1/DAAOko;60D04GAL4/+*, *UAS-TrpA1/+;11H05GAL4/SRkoGAL4*, and *UAS-TrpA1/+;60D04GAL4/SRkoGAL4* flies at both 22° and 29°C, with consolidated sleep data from day 1 and day 2 at each temperature. (**E**) Boxplot statistics for the sleep amounts of *11H05GAL4*- and *60D04GAL4*-associated flies at 22° and 29°C. Significance labels are omitted. Data are presented as medians with the 25th and 75th percentiles. Statistical analysis was performed using the pairwise Wilcoxon test (C and E). Relevant statistical information can be found in data S1. h, hours; n.s., not significant.

The activation of wake-promoting neurons labeled by *11H05GAL4* or *60D04GAL4* using the heat-activated cation channel TrpA1 at 29°C resulted in a significant reduction in daily sleep and an accumulation of gut ROS in flies (*16*, *36*). To investigate the function role of d-Ser in SD-induced gut ROS accumulation, we used above model while simultaneously manipulating the expression of genes relevant to d-Ser (Fig. 1A). The following fly lines were used for genetic recombination within the model: *SR* knockout (*SRko*), *SRkoGAL4* (where the coding region is replaced by the *GAL4* gene), *SRkiGAL4* (where *GAL4* is fused to *SR*), *CG12338* knockout (*CG12338ko*), and d-amino acid oxidase knockout (*DAAOko*), which is a double knockout of the *CG11236* and *CG12338* genes (*29*).

Initially, we introduced *CG12338ko* or *DAAOko* to *11H05* and *60D04 lines* and cross them with *TrpA1* to potentially reduce d-Ser degradation capacity. Additionally, we introduced *SRko* or *SRkoGAL4* to *TrpA1 line* and cross them with *11H05* or *60D04*, aiming to reduce d-Ser production capacity. Results from quantitative polymerase chain reaction (qPCR) experiments indicated that the genetic manipulations successfully reduced *SR* and *DAAO* mRNA expression levels, respectively (fig. S1A).

Before assessing whether d-Ser influences ROS accumulation in the gut, we first needed to determine whether genetic manipulations affected the extent of SD. Knockout of *SR* in the parental lines resulted in decreased nighttime sleep, while knockout of *DAAO* led to increased sleep at 22°C (Fig. 1, B to C; and fig. S1, B to C). These findings are consistent with the known roles of *SR* and *DAAO* in sleep regulation (*29*). Additionally, raising the environmental temperature to 29°C further reduced sleep across most parental lines, consistent with classical behaviors observed in flies (Fig. 1, B to C; and fig. S1, B to C) (*37*).

The hybridization process yielded *SRko/+* flies (*11H05*>*TrpA1; SRko/+* or *60D04*>*TrpA1; SRko/+*, referred to as *SR* heterozygotes), *SRkoGAL4/+* flies, *CG12338ko/+* flies, and *DAAOko/+* flies. These lines did not show significant changes in sleep amounts at 22°C but exhibited a substantial reduction in daily sleep at 29°C (Fig. 1, D to E; and fig. S1, D to E), indicating that the knockdown of *SR* or *DAAO* did not mitigate the effects of sleep loss when TrpA1 was expressed in neurons labeled by *11H05* or *60D04*.

It is important to note that *SR* is expressed in four neurons in the brain, in four pairs of neuronal tracts projecting to the ventral nerve cord (VNC), and in the midgut ECs (*29*). For the *SRkoGAL4/+* flies, it can be reasonably assumed that all neurons expressing *SR* would also be activated by TrpA1 at 29°C. To investigate the specific role of SR activation during SD, we generated *TrpA1; SRkiGAL4* flies and monitored their sleep at 29°C. The *TrpA1; SRkiGAL4* and *TrpA1; SRkoGAL4* flies retained sleep at 29°C, indicating that the activation of *SR*-expressing cells did not induce SD (fig. S1, B and C).

**Fig. 2.**
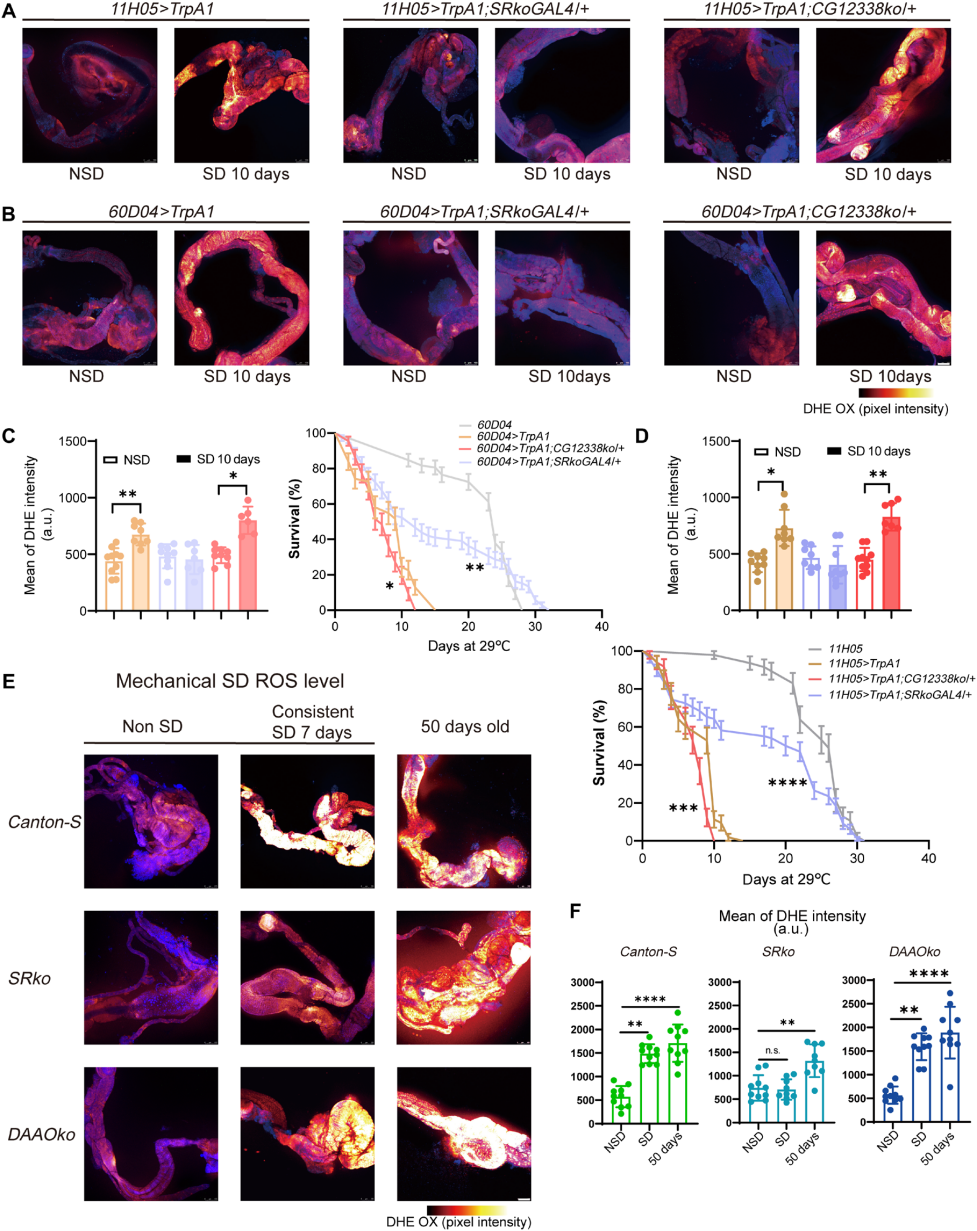
Fruit flies with *SR* knockout show reduced accumulation of gut ROS and an extended life span during SD. (**A** and **B**) Representative confocal images of the gut displaying oxidized DHE (DHE OX) in flies subjected to thermogenetic SD for 10 days. Scale bars, 100 μm; pseudo-color “glow” has been applied. (**C** and **D**) Quantification of DHE intensity and survival rates of flies undergoing thermogenetic SD. *60D04GAL4* and *11H05GAL4* lines were used a parental control. (**E**) Representative confocal images of the gut showing DHE OX in flies that experienced consistent mechanical SD for 7 days, compared to 50-day-old flies that did not undergo SD. Scale bars, 100 μm; pseudo-color “glow” has been applied. (**F**) Quantification of DHE intensity from the images in (E). Data are presented as means ± SEM. Statistical analyses were conducted using the Kruskal-Wallis test with Dunn’s multiple comparisons test (C, D, and F) or the log-rank test (C and D). n.s., not significant; **P < 0.01; ***P < 0.001; ****P < 0.0001. Relevant statistical information can be found in data S1. a.u., arbitrary unit.

To further evaluate the functional relationship between d-Ser and gut ROS accumulation during SD, we assessed the life span and gut ROS levels of the aforementioned fly lines following 10 days of thermogenetic SD. Notably, life span was significantly shorter for *CG12338ko/+*-deprived flies compared to control lines (*11H05*>*TrpA1* or *60D04*>*TrpA1*). Moreover, *DAAOko/+* flies exhibited the shortest life span among all genotypes tested. In contrast, the *SRko/+*-deprived flies showed a longer life span, with the *SRkoGAL4/+*-deprived flies demonstrated even greater longevity compared to the *SRko/+* flies (Fig. 2, A to D; and fig. S2, A to B).

To eliminate potential confounding effects from GAL4 activation of *SR*-expressing neurons, we also evaluated the life span of *SRkiGAL4/+* flies and found that thermogenetic SD led to premature death in these flies (fig. S2, A to B). These results suggest that the activation of *SR*-expressing cells does not enhance life span during SD; rather, the extended life span observed is likely due to the absence of d-Ser. Additionally, the knockout of d-Ser–related genes did not affect the longevity of the parental generation (fig. S2C).

To specifically assess gut ROS accumulation during thermogenetic SD, we used dihydroethidium (DHE), a sensitive and specific probe for superoxide radicals, to measure ROS levels in flies subjected to 10 days of SD (*38*). Elevated temperatures did not affect gut ROS levels in the parental control lines that were not subjected to SD (fig. S2D). However, due to the accelerated mortality in *DAAOko/+*-deprived flies, it was difficult to obtain a sufficient number of survivors for daily ROS measurements on day 10. Instead, we analyzed ROS levels using *CG12338ko/+* flies. Consistent with the life span phenotype, *CG12338ko/+* flies exhibited elevated gut ROS levels, while *SRkoGAL4/+* flies did not accumulate ROS after 10 days of SD compared to the *11H05*>*TrpA1* or *60D04*>*TrpA1-*deprived flies (Fig. 2, A to D, and fig. S2E).

Additionally, we assessed gut ROS accumulation after 4 days of thermogenetic SD. It was observed that 4 days of SD did not induce significant changes in DHE levels in the guts of *60D04*>*TrpA1* and *11H05*>*TrpA1* flies. Conversely, the knockdown of *DAAO* resulted in a significant increase in DHE levels, indicating that *DAAO* knockdown accelerated gut ROS accumulation in response to SD (fig. S2, F and G). To sum up, our findings confirm that gut d-Ser is necessary for ROS accumulation in flies subjected to thermogenetic SD.

In contrast to the TrpA1 activation observed in cholinergic neurons, the *11H05* and *60D04* neurons were found to bypass the sleep homeostasis mechanism without inducing compensatory sleep (*36*). However, prolonged SD often leads to a significant accumulation of sleep debt, thereby reactivating the sleep homeostasis mechanism. To investigate this further, we used an SD method using mechanical vibration, as it reflects sleep homeostatic mechanisms (*39*). This approach also enabled direct assessment of homozygous knockout mutants of d-Ser–related genes to validate our findings.

Mechanical vibration induced comparable SD effects on the first and seventh days of treatment (fig. S3, A to B). After 7 days of sustained mechanical SD, gut ROS levels were not significantly increased in *SRko*-deprived flies, while a marked increase was observed in *DAAOko*-deprived flies compared to wild-type *Canton-S* (Fig. 2, E and F). To rule out the possibility that the observed effects were influenced by factors unrelated to the genes in question, we measured gut ROS levels in flies subjected to discontinuous vibration [only during zeitgeber time 12 (ZT12)–ZT24, allowing for 12 hours of sleep recovery, with total vibration time equal to that of continuous vibration]. During these conditions, all genotypes exhibited no significant ROS accumulation (fig. S3, C and D).

Additionally, we also assessed ROS levels in the guts of aged flies to exclude the possibility of a deficiency in ROS production capability. The results indicated that the deficiency of d-Ser metabolic enzymes did not impair the ability of cells to produce ROS across all genotypes of 50-day-old flies (Fig. 2, E to F; and fig. S3, C to D). In conclusion, the findings presented in this section demonstrate that d-Ser, synthesized by SR and eliminated by DAAO, is essential for the accumulation of gut ROS in organisms subjected to prolonged SD.

### Treatments and analyses support gut-derived d-Ser’s role in gut ROS after SD

**Fig. 3.**
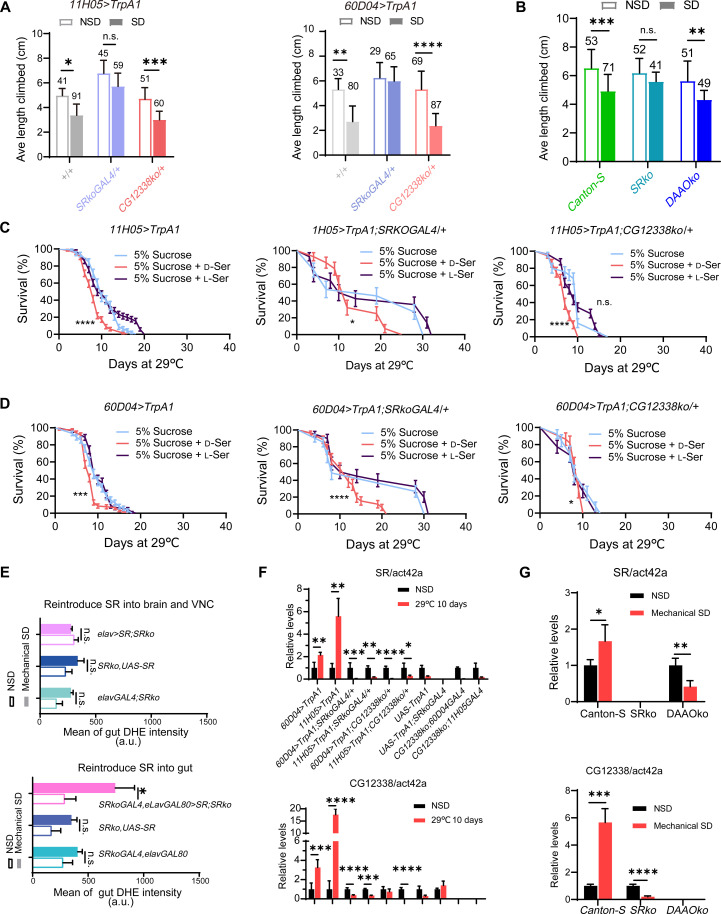
Increased levels of d-Ser lead to gut ROS accumulation during SD. (**A** and **B**) The negative geotaxis assay indicates that flies lacking d-Ser after SD exhibit significantly greater overall muscle and neuronal functions. The numbers indicate the sample size. (**C** and **D**) Survival rates of thermogenetic sleep-deprived flies fed 5% sucrose food supplemented with l-Ser or d-Ser. (**E**) Reintroduction of *SR* expression in the gut, but not in the brain, induces gut ROS accumulation during SD. Genotypes:*elavGAL4;SRko*, *SRko,UAS-SR*, *elavGAL4/+;SRko/SRko,UAS-SR*, *SRkoGAL4,elavGAL80*, *SRkoGAL4,elavGAL80*/*SRko,UAS-SR*. a.u., arbitrary unit. (**F** and **G**) Relative mRNA expression levels of *SR* and *CG12338* in sleep-deprived flies. Data are presented as means ± SEM. Statistical analyses were performed using the Kruskal-Wallis test with Dunn’s multiple comparisons test (A and B), a two-sample *t* test with Bonferroni-Dunn correction (F and G), the Mann-Whitney *U* test (E), or the log-rank test (C and D). n.s., not significant; *P < 0.05; **P < 0.01; ***P < 0.001; ****P < 0.0001. Relevant statistical information can be found in data S1.

Overall muscle and neuronal functions serve as indicators of the health status of flies subjected to SD, which has been shown to lead to decreased reaction speeds and even the onset of mental disorders (*40*). To evaluate these effects, we assessed climbing ability after 10 days of thermogenetic SD or 7 days of mechanical SD. As expected, SD resulted in a decline in the overall muscle and neuronal functions in control flies (Fig. 3, A and B). In contrast, *DAAOko*- and *DAAOko/+*-deprived flies exhibited even greater deterioration in climbing ability (Fig. 3, A and B). Additionally, the 10-day heat treatment did not affect the climbing ability of the parental control flies (fig. S4A). These findings suggest that flies lacking d-Ser maintained better muscle and neuronal responses when subjected to SD compared to those with functional d-Ser pathways.

To further support the role of d-Ser, we investigated the effects of dietary supplementation with a sucrose medium containing either d-Ser or l-Ser on the flies. We found that continuous consumption of d-Ser led to the rapid demise of the flies, regardless of whether they were sleep deprived. In contrast, l-Ser had no impact on life span (Fig. 3, C to D, and fig. S4B). Additionally, we assessed gut ROS levels in *SRkoGAL4/+* flies fed a sucrose medium containing either l-Ser or d-Ser after 7 days of thermogenetic SD. The results indicated that exogenous d-Ser supplementation, but not l-Ser, resulted in gut ROS accumulation in the *SRkoGAL4/+*-deprived flies (fig. S4, C and D). These results suggest that elevated levels of d-Ser are detrimental to fly health.

To determine whether specific tissue regions express *SR* and influence gut ROS accumulation, we reintroduced *UAS-SR* into various tissue within the *SRkoGAL4* background. Mechanical vibration produced comparable SD effects across different genotypes (fig. S5, A and B). The reintroduction of *SR* into brain cells did not elicit a gut ROS phenotype similar to that observed in wild-type flies after mechanical SD. In contrast, reintroducing *SR* into the gut ECs resulted in the accumulation of gut ROS (Fig. 3E), aligning with the findings that the gut is the primary organ affected by oxidative stress during SD.

Abnormal levels of d-Ser in the gut are detrimental to the health of flies and may remain elevated during SD. To investigate this hypothesis, we measured the mRNA levels of *SR* and *DAAO* in the gut across various genotypes under treated and untreated conditions. The qPCR data revealed a significant up-regulation of both gut *SR* and *DAAO* mRNA levels during SD, suggesting that high levels of d-Ser in circulation may arise from increased production by SR and catabolism by DAAO (Fig. 3, F and G). In the *SRkoGAL4/+*-deprived flies, *DAAO* mRNA levels in the gut were down-regulated, as there was no need for *DAAO* overexpression to degrade d-Ser due to the knockdown of *SR*. Conversely, the *CG12338ko/+*- and *DAAOko/+*-deprived flies regulated their ECs to decrease d-Ser production, resulting in a significant down-regulation of *SR* mRNA (Fig. 3, F and G). Together with the previous data on ROS, these findings suggest that elevated levels of d-Ser in the gut contribute to accelerated turnover during SD.

### ROS accumulation resulting from nighttime EC activation requires gut *SR*’s expression

**Fig. 4.**
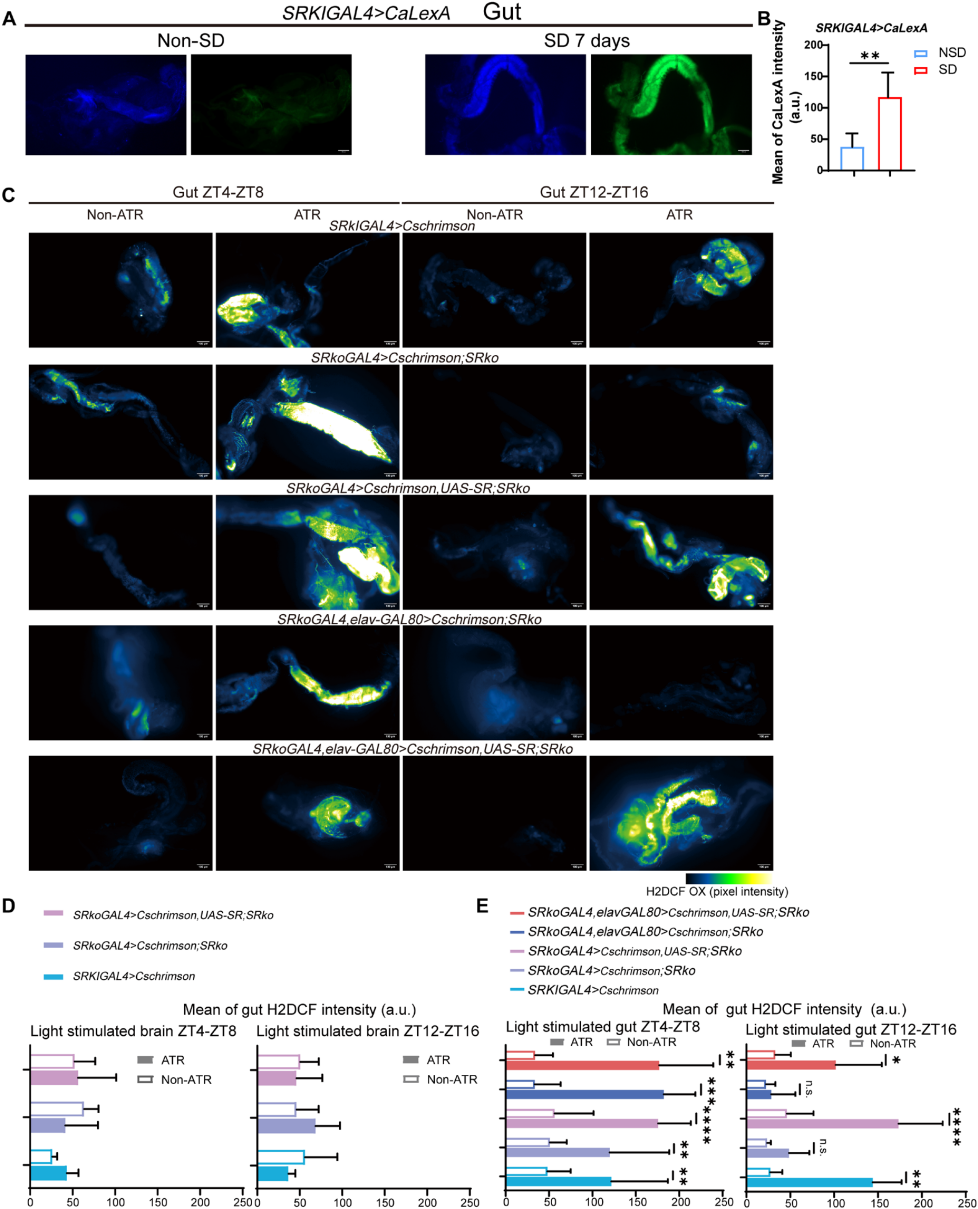
ECs activated by Cschrimson require gut d-Ser to induce ROS accumulation during the night phase. (**A**) Representative DAPI and GFP fluorescent images of the gut from SD and non-SD *SRkiGAL4*>*CaLexA* flies. Scale bars, 100 μm. (**B**) Quantification of GFP intensity from the images in (A). (**C**) Representative fluorescent images of gut H2DCF in ATR-fed and non–ATR-fed flies illuminated on the head or abdomen during the time intervals ZT4-ZT8 or ZT12-ZT16. Scale bars, 100 μm; the pseudo-color “Green Fire Blue” has been applied. (**D** and **E**) Quantification of gut H2DCF intensity. Genotypes: *UAS-Cschrimson,mcherry/+;SRkiGAL4/+*, *UAS-Cschrimson,mcherry/+;SRkoGAL4/SRko*, *UAS-Cschrimson,mcherry/+;SRkoGAL4/SRko,UAS-SR*, *UAS-Cschrimson,mcherry/+;SRkoGAL4,elav-GAL80/SRko*, and *UAS-Cschrimson,mcherry/+;SRkoGAL4,elav-GAL80/SRko,UAS-SR*. Data are presented as means ± SEM. Statistical analyses were conducted using *t* tests with Bonferroni-Dunn correction (B) and the Mann-Whitney *U* test (D and E). n.s., not significant; *P < 0.05; **P < 0.01; ***P < 0.001; ****P < 0.0001. Relevant statistical information can be found in data S1. a.u., arbitrary unit.

We anticipated that various cell types, including ECs, would exhibit increased activity in response to the SD regimen, as the asleep/restricted state transitioned to an awake/active state. To investigate this, we used *SRkiGAL4* to express a transcriptional reporter system based on a calcium-dependent nuclear import of LexA [Nuclear factor of activated T cells (NFAT)]–based tracing methodology (CaLexA) to determine whether ECs could be activated by SD (*41*). In this system, green fluorescent protein (GFP) expression indicated sustained cell activity (*42*). Continuous mechanical SD over a 7-day period resulted in significant increased GFP signal in the gut, but not in the brain or VNC of *SRkiGAL4*>*CaLexA* flies (Fig. 4, A to B; and fig. S6, A to B). The repeated experiments using another EC driver *mex1GAL4* showed that ECs were also activated by SD (fig. S6, C and D). These results demonstrate that ECs are trapped in a state of continuous activation due to SD, raising the question of whether this persistent activation of *SR*-expressing cells is sufficient to induce ROS accumulation in the gut.

To explore this further, we used optogenetics using the light-activated ion channel *UAS-CsChrimson.mCherry* to activate *SR*-expressing cells in specific tissues of flies fed all-trans-retinal (ATR) (*41*). Given the potential influence of circadian rhythm on *SR* expression, we implemented a two-time period strategy for activation. Flies were stimulated during ZT4-ZT8 (representing low sleep pressure and active state) and ZT12-ZT16 (representing high sleep pressure and transition to sleep) by shining a 590-nm laser specifically on the heads or abdomens of ATR-fed or ATR-unfed flies (*43*, *44*).

Due to the emission wavelength of the mCherry element in the flies coinciding with that of DHE, we used the green fluorescent hydrogen peroxide probe H2DCF to measure ROS levels (*45*). *SRkiGAL4*>*CsChrimson* flies that were fed ATR and illuminated in the abdomen, as opposed to the head, showed significantly elevated gut H2DCF intensity during both ZT4-ZT8 and ZT12-ZT16. These suggest that direct stimulation of the gut markedly enhances ROS levels, while activation of brain *SR*-expressing neurons has no significant effect on ROS levels (Fig. 4, C to E).

To further demonstrate the necessity of d-Ser for ROS accumulation induced by EC activation, we introduced *CsChrimson* into the *SRko* or *SRko,UAS-SR* background, driven by *SRkoGAL4*. In ATR-fed flies with the *SRkoGAL4*>*CsChrimson;SRko* genotype, illuminating the abdomen resulted in low gut H2DCF intensity during ZT12-ZT16. This low intensity was restored by the reintroduction of *SR* (*SRkoGAL4*>*CsChrimson;SR*). In contrast, illuminating the brain of ATR-fed flies did not increase gut H2DCF intensity, regardless of *SR* presence (Fig. 4, C to E).

To confirm that the observed effect was due to *SR* expression in the gut rather than the brain, we used a tissue-specific expression driver, *SRkoGAL4*, combined with *elav-Gal80*, to express *CsChrimson*. This approach allowed us to activate only nonneuronal *SR*-expressing cells using red light. Consistently, gut ROS accumulation was absent, as indicated by low H2DCF levels in nonneuronal *SR*-expressing cells when ATR-fed flies were illuminated in the abdomen during ZT12-ZT16; this accumulation could be restored by reintroducing *UAS-SR* (Fig. 4, C to E). Notably, all ATR-unfed flies exhibited minimal ROS levels following illumination of the head or abdomen, confirming that our optogenetic approach did not adversely affect the flies (Fig. 4, C to E). To conclude this section, our results demonstrate that ECs are overactivated by SD, leading to an excessive d-Ser release in response to heightened sleep demands, which ultimately results in gut ROS accumulation.

### The gut receives and integrates GABA and cholinergic signals to synthesize d-Ser

The objective of this phase was to determine whether ECs receive upstream signaling inputs that promote the expression of d-Ser. Disruption of these signals may lead to extended life spans in sleep-deprived flies. Recent studies have identified a sleep-regulating loop between the sleep homeostat and the circadian clock, involving key neural networks such as ellipsoid body R5 neurons (labeled by *69F08GAL4*), which track sleep pressure and encode sleep signaling output (*39*, *43*). Additionally, dFB neurons labeled by *23E10GAL4* have been shown to play a role in sleep homeostasis and promotion (*26*, *46*, *47*). It is hypothesized that sleep homeostat neurons communicate with ECs through the release of neurotransmitters (*21*, *48*, *49*).

**Fig. 5.**
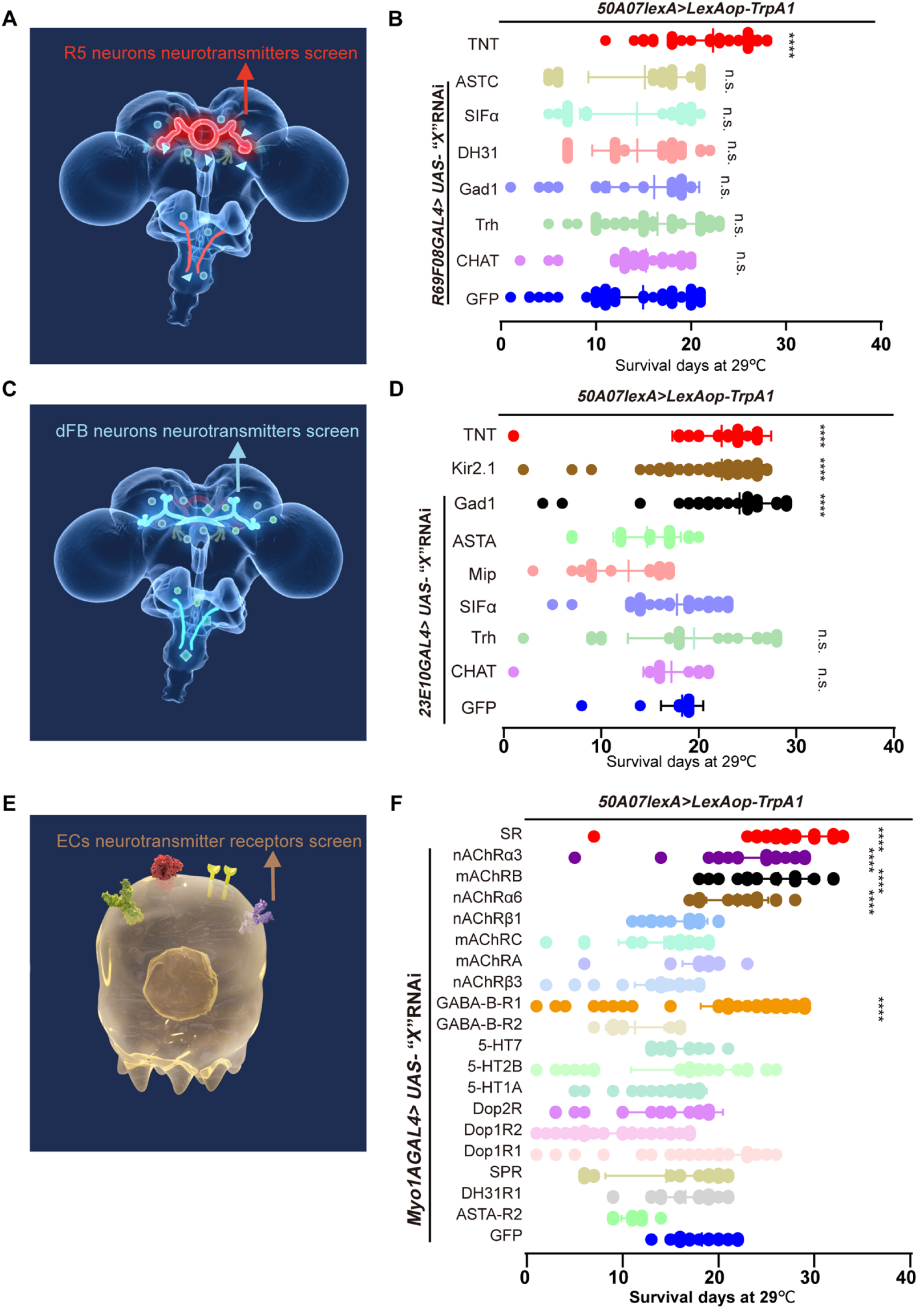
Gut GABA-B-R1 in ECs receives GABA signal input from dFB neurons. (**A**) Schematic diagram of the RNAi screening aimed at identifying neurotransmitters released by R5 neurons that can extend the life span of sleep-deprived flies. TNT, tetanus toxin; ASTC, allatostatin C; SIFa, neuropeptide SIFamide; DH31, diuretic hormone 31; Gad1, glutamic acid decarboxylase 1; Trh, tryptophan hydroxylase; CHAT, choline acetyltransferase. (**B**) Statistical plots of survival days indicate that R5 neurotransmitters do not extend life span. Kir2.1, inwardly rectifying potassium channel; ASTA, allatostatin A; Mip, myoinhibiting peptide precursor. (**C**) Schematic diagram of the RNAi screening for neurotransmitters released by dFB neurons that can extend the life span of sleep-deprived flies. (**D**) Statistical plots of survival days show that only GABA from dFB neurons extends life span. (**E**) Schematic diagram of the RNAi screening for neurotransmitter receptors in ECs that can extend the life span of sleep-deprived flies. (**F**) Statistical plots of survival days demonstrate that RNAi targeting GABA, CHAT receptors, and *SR* can extend life span. AChR, acetylcholine receptor; SPR, sex peptide receptor; GABA, γ-aminobutyric acid; 5-HT, 5-hydroxytryptamine (serotonin); Dopa, dopamine. Data are presented as means ± SEM. Statistical analyses were performed using the log-rank test (B, D, and F). n.s., not significant; ****P < 0.0001. Relevant statistical and genotypes information can be found in data S1.

To investigate the role of R5 and dFB neurons in upstream signaling and identify the neurotransmitters involved, we used two separate transgenic expression systems: *50A07LexA*>*LexAop-TrpA1*, which exhibit similar phenotypes to *GAL4s* in response to SD, to achieve thermogenetic SD and RNA interference (RNAi) driven by *69F08GAL4* or *23E10GAL4*, given their efficacy in conveying sleep needs. First, inhibiting neurotransmission by expressing UAS–tetanus toxin (*UAS-TNT*) in R5 or dFB neurons (*50A07*>*TrpA1;69F08*>*TNT* or *50A07*>*TrpA1;23E10*>*TNT*) significantly prolonged the life span of sleep-deprived flies (Fig. 5, B and D). These findings confirm that sleep homeostatic neurons modulate sleep-associated gut homeostasis. Furthermore, inhibiting γ-aminobutyric acid (GABA) synthesis in dFB neurons also resulted in a significant life-span extension (Fig. 5D). Notably, none of the neurotransmitters expressed in R5 neurons were identified in the screening (Fig. 5B).

Additionally, we conducted a screening of RNAi-mediated knockdown of neurotransmitter receptors in ECs to assess their ability to receive sleep-related signals. All receptors were expressed in ECs based on a single-cell RNA sequencing dataset (*50*). Unexpectedly, inhibiting GABA-B-R1 synthesis in ECs significantly prolonged the life span of sleep-deprived flies, while inhibiting another GABA receptor subtype (GABA-B-R2) did not show a similar effect (Fig. 5F). Moreover, RNAi targeting multiple acetylcholine receptor subtypes in ECs also extended the life span of sleep-deprived flies (Fig. 5F), suggesting that ECs can receive cholinergic sleep signals as well.

To provide further validation of the potential role of GABA signaling between dFB and ECs, the gut ROS levels in flies expressing Gad1-RNAi in dFB neurons were determined. The results demonstrated that the knockdown of Gad1 in dFB neurons resulted in a lack of gut ROS accumulation in SD flies (fig. S7, A and B). This approach was also applied to investigate whether *SR*-RNAi in ECs replicated the phenotypes observed in the above experiments of this work. Consistently, *SR* RNAi in ECs of sleep-deprived flies extended life span without increasing ROS levels (Fig. 5F and fig. S7, C to D), supporting our initial findings. No significant differences in sleep duration were observed at 29°C among the tested lines (fig. S8, A to C). These findings indicate that ECs receive and integrate at least GABA and cholinergic signals that influence sleep needs.

### Communication via d-Ser to NMDAR1 regulates intestinal ROS accumulation during SD

**Fig. 6.**
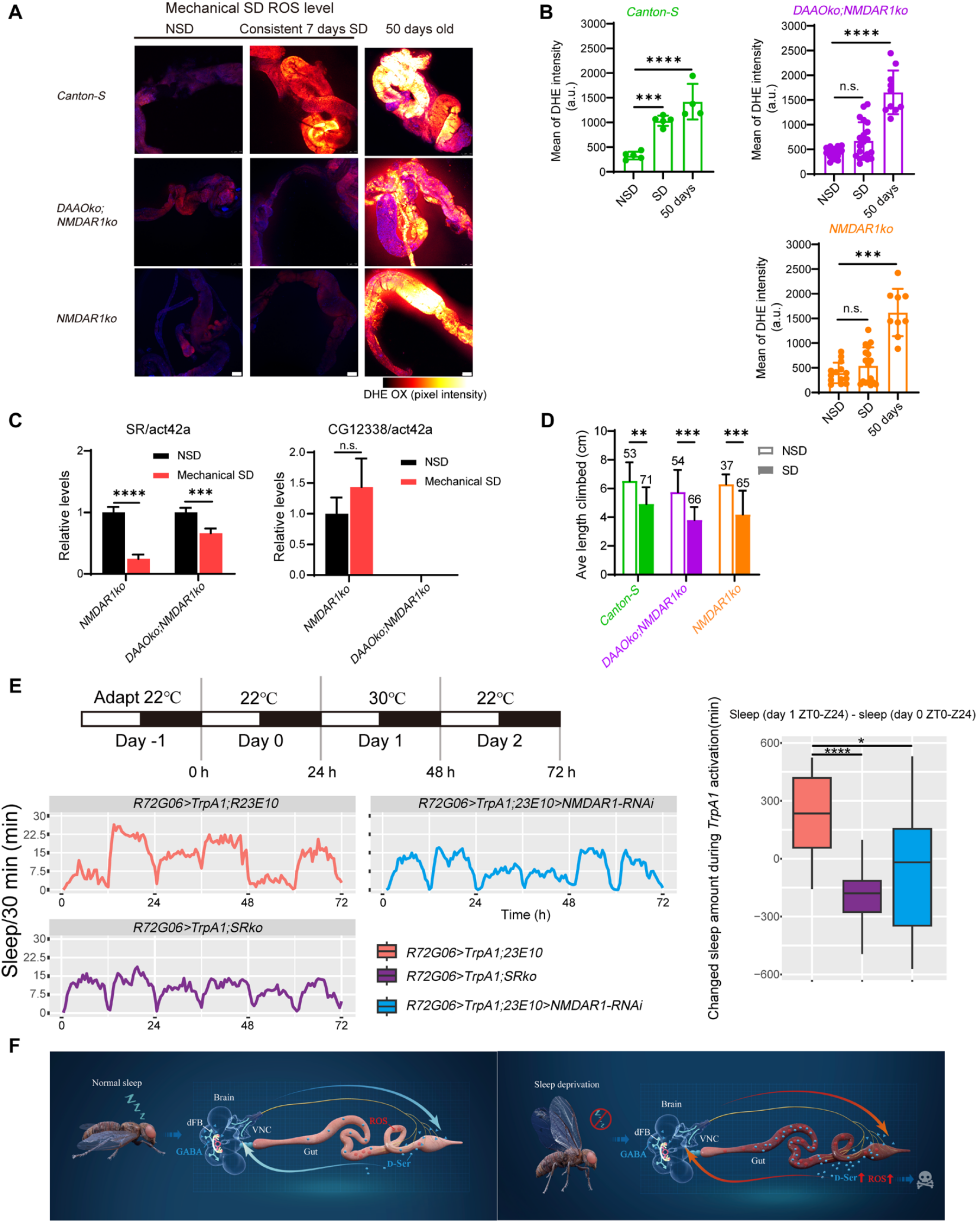
Downstream *NMDAR1* receives gut d-Ser signals to alleviate sleep pressure. (**A**) Representative confocal images of the gut displaying oxidized DHE (DHE ox) in wild-type *Canton-S*, *NMDAR1* knockout (*NMDAR1ko*), and *DAAO* knockout;*NMDAR1* knockout (*DAAOko;NMDAR1ko*) flies subjected to consistent mechanical SD for 7 days, as well as in 50-day-old flies that had not experienced SD. (**B**) Quantification of DHE intensity from (A). (**C**) Relative mRNA expression levels of SR and CG12338 in *NMDAR1ko* and *DAAOko;NMDAR1ko* flies subjected to consistent mechanical SD. (**D**) Negative geotaxis assay results indicate that sleep-deprived flies lacking *NMDAR1* exhibit poorer overall muscle and neuronal function. Sample sizes are indicated numerically. The data for *Canton-S* are the same as shown in Fig. 3B. (**E**) Enhanced sleep during stimulation of the *R72G06LexA* driver also necessitates *NMDAR1* in dFB neurons and SR. Genotypes: *R72G06LexA/+;LexAop-TrpA1/23E10GAL4*, *R72G06LexA/LexAop-TrpA1;SRko/SRko*, and *R72G06LexA/UAS-NMDAR1-RNAi;LexAop-TrpA1/23E10GAL4*. h, hours. (**F**) The schematic representation illustrates the potential brain-gut axis connections implicated in the regulation of SD-induced intestinal ROS accumulation by d-Ser. Data are presented as means ± SEM or as medians with the 25th and 75th percentiles. Statistical analyses were performed using the Kruskal-Wallis test with Dunn’s multiple comparisons test (B and C), *t* tests with Bonferroni-Dunn correction (D), and the pairwise Wilcoxon test (E). n.s., not significant; *P < 0.05; **P < 0.01; ***P < 0.001; ****P < 0.0001. Relevant statistical information can be found in data S1.

Upstream signaling input received by gut ECs has now been identified. However, it is reasonable to propose that there exists a feedback pathway where gut-derived d-Ser signals influence sleep pressure regulation. Specifically, NMDAR1, located downstream, facilitates nighttime sleep and alleviates sleep demand by receiving gut-derived d-Ser (*29*). *NMDAR1* is widely expressed in the brain, the VNC neurons, and the enteric nervous system (*29*). To determine whether gut ROS accumulation is regulated by NMDAR1, we examined the gut ROS phenotypes of *NMDAR1* knockout (*NMDAR1ko*) and *DAAO* knockout;*NMDAR1* knockout (*DAAOko*;*NMDAR1ko*) flies under SD conditions. The *NMDAR1ko* flies exhibited a significant reduction in their daily sleep patterns when maintained at 22°C (fig. S9A). The knockout of *NMDAR1* did not lead to an increase in gut ROS accumulation; instead, it unexpectedly reversed the substantial rise in ROS levels caused by *DAAOko* during SD (Fig. 6, A and B). SD significantly decreased the gut SR mRNA levels in *NMDAR1ko* flies but did not affect the gut *DAAO* mRNA levels (Fig. 6C). This suggests that NDMAR1 is necessary for promoting sleep to mitigate the severe sleep pressure induced by continuous SD, which also depends on d-Ser for co-activation. In contrast to the observed low levels of gut ROS, *NMDAR1ko* flies exposed to sleep pressure displayed markedly poor climbing ability (Fig. 6D). Although the knockout of these genes did not result in any observable impact on life span under non-SD (NSD) conditions at 22°C (fig. S9B), this finding aligns with the established role of NMDAR in neurogenesis and signaling in both *Drosophila* and mammals, including its crucial role in synaptic plasticity for learning, cognition, and memory (*51*, *52*). We also tested whether feeding d-Ser affects the gut ROS levels of *NMDAR1ko* flies. The results indicated that, regardless of SD or normal sleep, the exogenous addition of d-Ser did not induce an accumulation of gut ROS in the *NMDAR1ko* flies (fig. S9C). These findings suggest that NMDAR1 may be a downstream target of d-Ser–induced gut ROS accumulation after SD.

To determine which specific subsets of *NMDAR1*-expressing neurons are involved in the activation of signals induced by SD, we focused on R5 ellipsoid body neurons, which track sleep pressure and encode sleep signaling output (*39*). Knockdown of *NMDAR1* in a subset of dFB neurons also significantly decreased sleep (*53*), suggesting that dFB neurons may be one of the target subsets. Therefore, our next objective was to determine whether NMDAR1 in these brain regions plays a role in receiving gut d-Ser signaling.

We used *NMDAR1*-RNAi driven by *69F08GAL4* or *23E10GAL4*, along with simultaneous activation of *R58H05LexA*, which labels R5 neurons, for 12 hours during the night to measure sleep patterns during the night and the following day (*39*, *54*). The *R58H05LexA* driver exhibited a transient duration of SD during the stimulation period, followed by rebound sleep (fig. S9D). This finding supports the hypothesis that inputs activating sleep homeostasis occur after periods of wakefulness (*26*). However, RNAi of *NMDAR1* in dFB neurons did not produce rebound sleep. In contrast to previously published data, RNAi of *NMDAR1* in the *69F08GAL4* driver still resulted in rebound sleep following stimulation. Next, we knocked out *SR* in the *R58H05*>*TrpA1* flies and measured rebound sleep. In the absence of *SR*, nighttime stimulation with the *R58H05LexA* driver effectively elicited rebound sleep (fig. S9D), consistent with previous studies showing that *SR* knockout mutants still exhibit rebound sleep (*29*). These results indicate that d-Ser does not promote sleep through NMDAR1 in R5 neurons, which is why the *R58H05LexA* driver can still induce rebound sleep in the absence of *SR*.

Subsequently, we activated *R72G06LexA*, which labels dFB neurons, for 24 hours to assess its sleep-promoting effects (*39*, *46*). The *R72G06LexA* driver showed a significant increase in sleep during the thermogenetic stimulation period. Notably, the absence of *NMDAR1* expression in dFB neurons or a genomic deletion of *SR* did not produce the sleep-promoting phenotype (Fig. 6E). Furthermore, flies expressing RNAi against *NMDAR1* in dFB neurons demonstrated a slight gut ROS accumulation and an extended life span during thermogenetic SD compared to *50A07*>*TrpA1;23E10*>*GFP* flies (fig. S10, A to C). However, the knockdown of *NMDAR1* in dFB neurons did not prevent the exacerbation of gut ROS accumulation after exogenous ingestion of d-Ser in SD flies (fig. S10, A and B). Collectively, these findings suggest that gut-derived d-Ser transmits sleep-related signals to downstream NMDAR1, with dFB neurons representing at least one of its target subsets. The ECs receive signaling inputs from upstream neurons, thereby influencing gut d-Ser biosynthesis and its activation of downstream NMDAR1, which, in turn, regulates sleep. The signal amplification that occurs because of SD may results in an imbalance in gut-brain communication, which, in turn, causes gut ROS accumulation (Fig. 6F).

### Abnormal gut d-Ser levels affect TCA cycle and peroxisome metabolism and cause ROS accumulation

Last, we aimed to investigate the impact of gut d-Ser on intestinal oxidative stress. To do this, we conducted metabolomic analyses on brain and gut tissues from flies sampled at ZT4-ZT8 to minimize potential circadian rhythm-related effects. The samples were categorized into two groups: (i) based on whether the genotype knocked down *SR* (*11H05*>*TrpA1;SRkoGAL4/+* versus *11H05*>*TrpA1* and *60D04*>*TrpA1;SRkoGAL4/+* versus *60D04*>*TrpA1*, *SRko* versus *Canton-S*) to assess how *SR* knockdown affects metabolism; and (ii) grouped by SD and NSD according to whether the flies underwent 10 days of thermogenetic SD or 7 days of mechanical SD, to examine the effects of SD on metabolic pathways.

A total of 4616 metabolites were identified in the gut samples, while 3758 metabolites were identified in the brain samples (fig. S11, A and B). The results demonstrated the significant up-regulation of antioxidant molecules, amino acids and sugar compounds in the brain of flies following SD, including glutamylalanine, taurine and lauric acid (fig. S12, A to C). Furthermore, it was observed that SD resulted in substantial alterations in the levels of various phosphatidylcholines, amino acids, and lipids within the gut of the flies (fig. S13, A to C). SR knockdown has been observed to enhance the levels of glycosphingolipids on ceramide and precursors for the synthesis of several neurotransmitters in the brain, such as *N*-acetyl-l-aspartate acid and *N*-acetylaspartylglutamic acid (fig. S14, A to C). Furthermore, SR knockdown has been observed to result in significant up-regulation or down-regulation of various phosphatidylcholines and acylcarnitines in the gut. In addition, SR knockdown has been observed to up-regulate the levels of 3,4-dimethyl-5-pentyl-2-furandecanoic acid and dodecanedioic acid in the gut, which are recognized for their protective effect against lipid peroxidation (fig. S15, A to C). Enrichment analysis revealed some similarities in the signaling pathways shared by the gut and brain. The identified pathway terms included “lysine degradation,” “tryptophan metabolism,” “sphingolipid metabolism,” and “phosphatidylinositol signaling system,” suggesting that various functions of both the gut and brain—such as signal transduction, membrane transport, and amino acid metabolism—are affected by SD. Additionally, the pathway terms included “Galactose metabolism,” “amino sugar and nucleotide sugar metabolism,” “starch and sucrose metabolism,” “fructose and mannose metabolism,” and “glycolysis or gluconeogenesis,” indicating that sugar metabolism in both the gut and brain is influenced by *SR* knockdown (fig. S16, A and B). Notably, fatty acid metabolism in the gut, but not in the brain, was significantly affected by the SD process or *SR* knockdown (fig. S16, A and B). This indicates that the metabolic changes triggered by SD are not entirely overlapping between the brain and gut and that gut fatty acid metabolism is likely influenced by d-Ser.

**Fig. 7.**
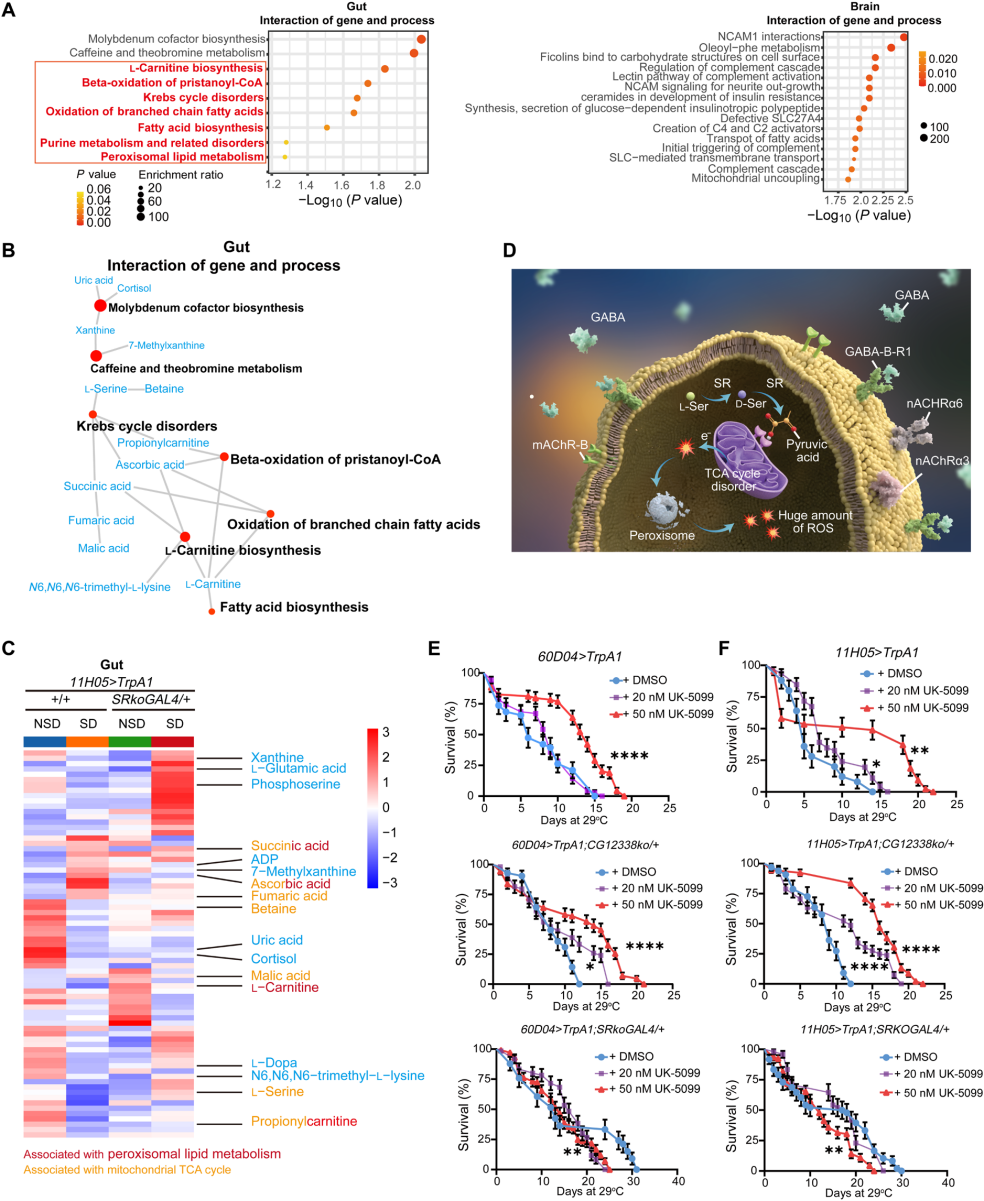
Gut d-Ser influences pyruvate levels to enhance ROS accumulation. (**A**) The bubble chart displays the top significant enrichment pathways for brain and gut samples, illustrating the interaction between SD and the knockdown of *SR*. NCAM1, Neural Cell Adhesion Molecule 1; SLC, solute carrier. (**B**) The interconnections of the enriched pathways and the metabolites identified in (A). The size of each circle corresponds to the significance of the enriched pathway, with differentially expressed molecules highlighted in blue. CoA, coenzyme A. (**C**) Simplified clustered heatmap showing changes in expression of gut metabolites hit in ASCA analysis of (*11H05*>*TrpA1;SRkoGAL4/+* versus *11H05*>*TrpA1*) group data. Differentially expressed molecules associated with lipid peroxide metabolism highlighted in red, and those associated with the mitochondrial TCA cycle highlighted in yellow. (**D**) Schematic diagram illustrating the mechanism by which gut d-Ser induces ROS accumulation in the gut. (**E** and **F**) Survival rates of thermogenetic sleep-deprived flies fed different concentrations of UK-5099. Data are presented as means ± SEM or as medians with the 25th and 75th percentiles. Statistical analyses were conducted using the log-rank test (E and F). n.s., not significant; *P < 0.05; **P < 0.01; ****P < 0.0001. Relevant statistical information can be found in data S1.

The interaction between two factors, SD and *SR* knockdown, was identified through data screening and modeling using the ASCA methodology (*55*). This analysis revealed which metabolic pathways altered by SD lead to gut ROS accumulation and how these pathways are modified by *SR* knockdown. The enrichment analysis of gut and brain samples yielded distinctly different results (Fig. 7A). The pathway terms identified in the gut samples included “Krebs cycle disorders,” “beta-oxidation of pristanoyl–coenzyme A,” “oxidation of branched chain fatty acids,” “fatty acid biosynthesis,” “peroxisomal lipid metabolism,” and “l-carnitine biosynthesis.” These findings suggest that the changes in gut function observed in response to SD, particularly alterations in the tricarboxylic acid (TCA) cycle within mitochondria and lipid metabolism in peroxisomes, may be influenced by *SR* knockdown (Fig. 7A).

Using a simplified metabolic network and cluster map, we illustrated the interconnections of enriched pathways and metabolites identified in the metabolomics data (Fig. 7, B and C). Flies subjected to SD exhibited significantly elevated levels of succinic acid and fumaric acid, indicating enhanced TCA metabolism with increased electron leakage within the respiratory chain. Conversely, SD flies showed a marked decline in propionylcarnitine levels, reflecting severe disruption of branched-chain amino acid metabolism and potential damage to peroxisomes (Fig. 7, B to C, and fig. S17A). Notably, sleep-deprived flies lacking *SR* did not exhibit significant changes in fumaric acid levels. Additionally, the *SR*-deficient flies remained significantly higher propionylcarnitine levels than those in the SD control group (Fig. 7, B to C, and fig. S17A).

Peroxisomes are highly dynamic organelles that directly regulate cellular lipid metabolism and ROS levels, which influence mitochondrial function (*56*). The precise causal relationship between the impairments of the TCA cycle and peroxisome metabolism caused by SD remains unclear. However, a potential hypothesis regarding the mechanism by which d-Ser affects the accumulation of ROS in the gut is as follows: Under SD conditions, d-Ser racemization from l-Ser and dehydration to pyruvate and ammonia by *SR* was accelerated. This increase in pyruvate may elevate TCA cycle activity, leading to notable electron leakage during electron transfer in the respiratory chain. Consequently, this would result in considerable ROS accumulation and ultimately impair peroxisomal function (Fig. 7D).

Pyruvate must first be transported into the mitochondria by the mitochondrial pyruvate carrier (MPC) before it can be used in the TCA cycle (*57*). To validate this hypothesis, sleep-deprived flies were fed a diet containing the MPC inhibitor UK-5099 (*58*), and their life spans were recorded. The administration of 20 nM UK-5099 significantly extended the life span of *CG12338ko/+* sleep-deprived flies, while no effect was observed in the control group. Furthermore, 50 nM UK-5099 markedly extended the life span of both control and *CG12338ko/+* sleep-deprived flies while reducing the life span of *SRkoGAL4/+*-deprived flies (Fig. 7, E and F). Consistent with these findings, after 7 days of SD, *11H05*>*TrpA1* flies fed 50 nM UK-5099 did not accumulate gut ROS (fig. S17B). These results indicate that limiting the influx of pyruvate into the mitochondria is an effective strategy to reverse the accelerated mortality associated with elevated gut d-Ser levels.

## DISCUSSION

Insomnia has emerged as a substantial concern in modern society, affecting both physical and psychological well-being. One of the most direct and serious consequences of insomnia is the induction of oxidative stress in the intestine (*12*, *16*). Our study’s findings suggest that an endogenous amino acid plays a crucial role in the accumulation of gut ROS associated with SD.

We have demonstrated that sleep loss induces the biosynthesis of d-Ser at both the transcript and amino acid levels. The role of d-Ser is further supported by evidence showing that the knockout mutant of *SR* (or *DAAO*) exhibited decreased (or increased) ROS accumulation, climbing ability, and life span. Notably, the combined action of wake-promoting neurons with the *SRkoGAL4* line resulted in the most pronounced phenotype, an extended life span. The key distinction between the *SRkoGAL4/+*- and *SRko/+-*deprived flies is that neurons and ECs labeled by *SRkoGAL4* were also activated by TrpA1.

Given the delayed mortality observed in *SRkoGAL4/+* flies and the lack of efficacy in the ROS rescue phenotype upon reintroducing *SR* into *SR*-expressing neurons (fig. S2, A to B, and Fig. 3E), it is reasonable to conclude that the additional effect may stem from the stimulation of ECs lacking *SR*. Supporting this conclusion, gut ROS production was induced solely by optogenetic stimulation of *SR*-expressing ECs, not neurons. However, the expression and activation of *TrpA1* in ECs (*Myo1A*>*TrpA1*) did not lead to any changes in gut ROS levels, as evidenced by other research studies (*16*).

This discrepancy can be attributed to two factors. First, the cluster of *SRGAL4*-labeled cells is a subset of *Myo1AGAL4*, comprising the majority of ECs in the midgut (*35*). Second, unlike photosensitive proteins that are expressed on cell membranes to activate cells in response to light, TrpA1 is an injury receptor that integrates various injury signals, including temperature, chemicals, pain, and inflammation, to modulate different outputs (*59*). Therefore, the activation effects may differ between these mechanisms.

What is the significance of enterogenic d-Ser levels for organisms with sleep requirements? Among the two isomers of serine, l-Ser can participate in the generation of oscillations of *S*-adenosylmethionine, which subsequently initiates the methylation of histones (*34*). There exists a reciprocal relationship between circadian rhythms and methyl metabolism (*60*). Moreover, the metabolisms of methylhistidine, methionine, and serine also exhibit rhythmic patterns (*60*–*63*). It has been reported that neural l-Ser metabolism inhibits sleep during periods of starvation, suggesting that ingestion and sleep are mutually antagonistic states (*27*). l-Ser in the brain is linked to both sleep and metabolism, and our studies suggest that d-Ser in the gut also affects sleep and metabolism. High gut d-Ser levels may disrupt intestinal homeostasis and lipid metabolism. This hypothesis will be investigated in the subsequent studies. Given the unique functions and compositions of the brain and gut, there may be unexplored biological significance in the different roles of these isomers across various organs. While neuronal circuits facilitate relatively rapid transitions between wake and sleep states, peripheral tissues such as the gut may undergo a more gradual process to reach a threshold that promotes sleep. The gut serves as the primary site for nutrient absorption, with the metabolic rate of macronutrients in the gut increasing after feeding and decreasing during the onset of sleep (*63*). To fall asleep, organisms must forgo the opportunity to eat, and gut cells do not need to be fully prepared for incoming nutrients (*64*, *65*). This “relaxation” signal should be transmitted to the brain to initiate sleep. Therefore, it is reasonable to propose that gut d-Ser metabolism may serve as this relaxation signal.

Our optogenetic experiments indicate that the accumulation of ROS induced by EC activation during the night phase is dependent on *SR* expression. This provides evidence for an *SR*-dependent mechanism of gut ROS accumulation in response to SD. Notably, the activation of ECs during the awake phase also leads to ROS production but does not require *SR* involvement. This phenomenon may be attributed to (i) the presence of a gating mechanism in mitochondria that responds to intracellular Ca^2+^ levels, with the response varying depending on the resting or active state of the cell (*66*); and (ii) the existence of *SR*-independent mechanisms that promote ROS increases during SD.

One of the key findings of this study is the pathway through which SD leads to increased d-Ser levels in the gut. The R5 neurons are particularly sensitive to the effects of sleep loss and encode neural signals that regulate sleep-driving processes (*39*). The dFB neurons play a crucial role in inducing sleep, influencing both the duration of sleep and sleep homeostasis (*54*, *67*). Notably, both R5 and dFB neurons are GABAergic and cholinergic (*43*, *46*).

Our data indicate that the life span of flies was extended following SD when GABA expression was knocked down in dFB neurons or GABA-B-R1 in the gut (Fig. 5, D and F). This effect may be attributed to the potential ineffectiveness of the sleep homeostasis driver under sleep pressure conditions. We hypothesize that GABA sleep need signaling from dFB neurons may transmit to GABA-B-R1 in the gut, activating ECs to biosynthesize d-Ser. If unresolved sleep stress persists, then ECs may continue to synthesize d-Ser as compensation. Additionally, ECs receive signaling inputs from various sources, as indicated by our screening results (Fig. 5F). The application of individual RNAi targeting multiple acetylcholine receptors significantly extends life span in sleep-deprived flies. Despite the fact that we were unable to complete the screening experiments using GAL4 drivers that are more specifically labeled with ECs, this will be a point to validate in future research. Notably, extensive research has been conducted on the role of different subtypes of acetylcholine receptors in regulating sleep and arousal (*68*). Acetylcholine neuronal connections between VNC and ECs have also been identified (*41*). The dFB neurons in the VNC appear to both receive brain signals and transmit peripheral signals to the brain (*46*). It is yet to be demonstrated whether dFB-VNC neurons have connections with ECs. Additionally, previous studies suggest that the activation of *11H05* or *60D04* neurons does not engage sleep homeostatic mechanisms, as evidenced by the absence of sleep rebound following heat activation (*16*, *36*). Notably, the results of this study imply the involvement of R5 and dFB neurons, although direct measurement of Ca^2+^ changes in sleep homeostatic neurons during thermogenetic SD had not been conducted in this study.

Our optogenetic experiments demonstrate that stimulating ECs during periods of elevated sleep demand, rather than during wakefulness, can lead to ROS accumulation (Fig. 4, D and E). It seems reasonable to suggest that there is a circadian oscillation in gut d-Ser levels, with a slight increase preceding sleep onset to facilitate sleep. In contrast, SD disrupts this oscillation, maintaining gut d-Ser at elevated levels. Thus, we propose that d-Ser serves as a critical physical indicator of accumulated sleep debt.

Furthermore, our results indicate that gut d-Ser may facilitate the release of accumulated sleep debt to the downstream receptor NMDAR1 (Fig. 6, A to E). Previous research has shown that dopamine transporter mutations (fmn) maintain normal gut ROS levels and life span even under chronic SD conditions (*23*). Additionally, fmn flies exhibited lower *NMDAR1* mRNA levels, as demonstrated by a screen of brain mRNA levels in these flies (*53*). Another study identified numerous differentially expressed genes related to ionotropic glutamate receptor binding, using spatial transcriptomics to explore the impact of SD across the brains of male mice (*69*). These findings further support our assertion that SR and NMDAR1 play crucial roles in sleep homeostasis and gut health.

How do gut d-Ser levels affect ROS accumulation caused by SD? d-Ser plays a crucial role in regulating glucose metabolism, exhibiting both beneficial and harmful effects in a dose-dependent manner. Chronic low doses of d-Ser have been shown to be beneficial in reducing weight gain induced by a high-fat diet (*34*). However, excessive accumulation of d-Ser can impair insulin secretion and lead to glucose intolerance (*33*). Additionally, insulin signaling in clock neurons has been demonstrated to regulate sleep patterns (*22*).

The results of the metabolomics study indicate that, in the SD or *SRko* alone conditions, there is a substantial alteration in the gut phosphatidylinositol signaling system, fatty acid metabolism, and sugar metabolism. However, our metabolomic data from the brain, but not the gut, indicated that the knockout of *SR* significantly altered the impact of SD on the synthesis and secretion of glucose-dependent insulinotropic polypeptide or glucagon-like peptide-1. This suggests that gut d-Ser is unlikely to induce gut inflammation through its effects on glucose metabolism (Fig. 7A and fig. S16).

It is important to note that the ASCA analyses performed in this study directly hit the TCA cycle in mitochondria versus lipid metabolism in peroxisomes in the gut rather than in the brain. Most intracellular ROS are generated from disturbances in the TCA cycle within the mitochondria and from imbalances in lipid metabolism in the peroxisomes (*70*–*73*). Peroxisomes are primary modulators of sensitivity to dietary nutrients, nutrient states, and ROS levels, producing antioxidant enzymes that combat inflammation. These results strongly suggest that gut d-Ser likely exerts a important influence on the vital functions occurring in the mitochondria and peroxisomes that result in ROS accumulation during SD. Furthermore, SR has been shown to facilitate the proliferation of colorectal cancer cells by enhancing the pyruvate pool and increasing mitochondrial ROS levels through the dehydration of serine (*35*). Given the elevated expression of *SR* and *DAAO* mRNAs in response to SD, the accumulation of gut d-Ser under sleep pressure may influence pyruvate levels (*73*, *74*). Metabolic disorders can affect peroxisome function and the activity of antioxidant enzymes, as evidenced by observations that SD can lead to lipid loss and glycogen depletion in the gut (*75*). Future research directions could, therefore, include investigating the effect of d-Ser on gut peroxisome and mitochondrial function and the causal relationship among the three under conditions of SD, resulting in ineffective scavenging of intestinal ROS. Moreover, it would be worthwhile to investigate whether specific dietary nutrient supplements could facilitate metabolic reprogramming and restore peroxisome functionality, potentially inhibiting ROS accumulation.

In conclusion, we have identified that SD-induced elevation of d-Ser biosynthesis in the gut plays a crucial role in intestinal ROS accumulation, potentially through gut-brain axis signaling via NMDAR1. Understanding the downstream effectors involved in this process will shed light on the molecular and neural connections between sleep and metabolism, presenting an intriguing avenue for future research.

## MATERIALS AND METHODS

### Drosophila melanogaster

All stocks were maintained on yeast-cornmeal-agar medium (2.5% w/v yeast, 10% w/v cornmeal, 5.5% w/v sucrose, 0.8% w/v ager, 1.6% v/v ethanol, 0.16% w/v methylparaben, and 0.4% v/v propionic acid) in the 12-hour:12-hour light:dark circulations at 22°C temperature and 60% humidity incubator. All experiments were performed using adult males. *SRko*-*Gal4*, *SRko*, *CG12338ko*, *DAAOko*, *NMDAR1ko*, *DAAOko*;*NMDAR1ko*, and *SRko,UAS-SR* were provided by Y. Rao (Chinese Institute for Brain Research, Beijing, China), and *UAS-Cschrimson,mCherry* were provided by G. Si (Chinese Academy of Sciences, Beijing, China). *50A07LexA* and *LexAop-TrpA1* were provided by W. Song (Wuhan University, Wuhan, China). RNAi lines were obtained from Bloomington Drosophila Stock Center (BDSC) or Vienna Drosophila Resource Center (VDRC).

### Sleep monitoring and locomotor activity

Sleep, locomotor activity, and feeding assay were achieved by ETHOSCOPE system (*76*). For ETHOSCOPE, the 5-day-old flies were loaded into glass tube, which had been injected with yeast-cornmeal-agar medium by using 1-ml syringe and adapted to this glass tube environment overnight. Raspberry Pi 3B and camera were used to record sleep and activity amount.

### Thermogenetic or mechanical vibration SD

Thermogenetic SD was achieved using the Gal4/UAS systems. Five-day-old male flies were loaded onto fresh vial and placed in a 29°C incubator to trigger SD. Mechanical SD was achieved using a multi-tube oscillator. Five-day-old male flies were loaded onto fresh tube and placed in oscillator. The multi-tube oscillators placed in a 22°C incubator and provided 2-s-long vibrations at random intervals per 1 min. The rotational speed of the oscillator was 1200 rpm. Incontiguous mechanical SD needed oscillators to work in ZT12-ZT24 and stop in ZT0-ZT12, vibration time every minute doubled.

### Gut ROS imaging

ROS imaging was used Owusu-Ansah’s or Alexandra’s protocols (*16*, *77*). Flies were anesthetized on ice and dissected in Schneider’s *Drosophila* medium (Gibco). DHE (Sigma-Aldrich) dissolved in dimethyl sulfoxide (DMSO) and pipetted 2 μl into Schneider’s *Drosophila* medium to a final concentration of 60 μM immediately. H2DCF (Sigma-Aldrich) dissolved in DMSO and pipetted 1 μl into 1× phosphate-buffered saline (PBS) to a final concentration of 10 μM immediately.

For DHE, intestinal tissues were immersed in the DHE and shaken at room temperature for 7 min in the dark and then washed and shaken in Schneider’s medium three times for 5 min at room temperature. Guts were mounted with Vectashield Antifade Mounting medium with 4′,6-diamidino-2-phenylindole (DAPI; Vector Laboratories) and imaged on an Olympus FV1000 or Leica DM6 confocal microscope. *Z* stacks were used to obtain whole-gut ROS.

For H2DCF, guts were immersed in the H2DCF and shaken at room temperature for 15 min in the dark and then washed and shaken in 1× PBS three times for 5 min at room temperature. Guts were mounted with Vectashield Antifade Mounting medium with DAPI and imaged on a modified Olympus ix73 fluorescent microscope. The mean of the summed pixel intensities of image was used to get gut ROS.

### Life-span analysis

Newly enclosed (6 hours) male flies were collected onto fresh vial with yeast-cornmeal-agar medium and then waited for growth to 5 days old in a 22°C incubator. After that, every stock of flies was maintained at a density of 10 to 25 flies per vial with different food medium in 22° or 29°C incubator. Flies were transferred to fresh medium every 2 days and counted the number of dead flies daily. For SD test, food was the yeast-cornmeal-agar medium described above. For exogenous supplementary d/l-Ser life-span test (Fig. 3, C to D, and fig. S4B), food was 5% sucrose and 0.8% agar containing d-Ser or l-Ser (2.9 g/liter; Tokyo Chemical Industry). For exogenous supplementary UK-5099 test, a stock solution of 1 mM UK-5099 was prepared using DMSO and then added to the yeast-cornmeal-agar food to the desired final concentration.

### Negative geotaxis assay

Negative geotaxis assay of flies was described by Nichols *et al.* (*40*). In brief, newly emerged adult male flies were collected into fresh food medium for 4 to 5 days at 22°C. After thermogenetic or mechanical vibration SD described above, 10 to 25 flies were transferred to the prepared polystyrene vials with transparent ruler at 22°C. Fifteen to 20 min later, the vials were sharply tapped three times to ensure all flies to the bottom. Then, a picture was taken by using a camera 3 s later. We let the flies rest for 1 min, and we repeated the procedure.

### Optogenetics experiments

ATR (Sigma-Aldrich, R2500) was added to yeast cornmeal-agar medium to a final concentration of 400 μM from a 50 mM ethanol stock. Male flies were reared in the dark and transferred to ATR diet or non-ATR diet 5 to 7 days after eclosion for feeding 2 days before experiments. Six flies were mounted under dim blue light in 35-mm glass-bottomed culture dishes (MatTek, P35G-0-10-C), in which flies were affixed by the polyvinyl acetate adhesive, Elmer’s Clear School Glue. A glass cover slide was used further immobilized flies. Mechanical microscope stepper stage drove 10× objective lens connected with a fiber optic that can emit 590-nm red laser created by a programmable lasers. Each flies’ head or abdomen were illuminated specifically for 4 s every 40 s, repeated 24 times, and every 30 min for 4 hours. Then, the flies were unmounted and dissected, H2DCF stained, and imaged immediately as described above.

### Liquid chromatography–tandem mass spectrometry untargeted metabolomics

Flies were anesthetized on ice and dissected in 1× PBS. About 250 flies’ brain and gut tissues were collected as one sample and rapidly frozen in liquid nitrogen and stored in a −80°C refrigerator. All samples were pretreated and injected into the Waters ACQUITY UPLC I-Class plus/Thermo QE HF ultrahigh-performance liquid tandem high-resolution mass spectrometer. All samples were guaranteed quality with quality control samples. Raw data were processed by the metabolomics processing software Progenesis QI v3.0 software (Nonlinear Dynamics, Newcastle, UK) and compared with the Human Metabolome Database, Lipidmaps (v2.3), and METLIN databases and the LuMet-Animal local database (Lu Ming Biotech). All data were analyzed with R (v4.3.3) and MetaboAnalyst (*53*).

### Quantitative PCR

Total RNA was extracted from ~60 dissected gut of flies using TRIzol reagent (Takara) and reverse transcripted using the PrimeScript RT reagent kit with guide DNA Eraser (Takara, RR047A). qPCR analysis was then performed using TB Green Premix Taq kit (Takara, RR420A) in the CFX96 real-time PCR detection system. The sequences of primers used are as follows: *SR*-F: 5′-CAGTGGCTATCCTAAGATTGGT-3′; *SR*-R: 5′-ACGGTGGTGTCTATGTTTCCT-3′; *act42a*-F: 5′-CTCCTACATATTTCCATAAAAGATCCAA-3′; *act42a*-R: 5′-GCCGACAATAGAAGGAAAAACTG-3′; *CG12338*-F: 5′-AACTCCAGAAGGAGTTTCCC-3′; *CG12338*-R: 5′-GGTCATCCATTGTTGC-GTAAT-3′; *CG11236*-F: 5′-ATACCCAACACCGAAAGTGT-3′; *CG11236*-R: 5′-TGCTCCAAACCAGGAATGTA-3′.

### Statistics and reproducibility

Data were analyzed using GraphPad Prism 8.0.2 (GraphPad) and R (v4.3.3). Sleep behavior data were analyzed by pairwise.wilcox.test. Two sets of independent samples were compared using Mann-Whitney *U* test. Multiple sets of measurements involving one independent variable were analyzed by Kruskal-Wallis test with Dunn’s multiple comparisons test. Survival data were analyzed by log-rank test. Data were expressed as means ± SEM. throughout, except in boxplots that show median ± interquartile ranges. Statistical significance was indicated as *P < 0.05, **P < 0.01, ***P < 0.001, and ****P < 0.0001.
