# Supplementary material for: Role of d-serine in intestinal ROS accumulation after sleep deprivation
Source: Sci Adv. 2025 Jul 18;11(29):eadr8592. doi: 10.1126/sciadv.adr8592 (PMC12273795; doi:10.1126/sciadv.adr8592)
Supplement: Supplementary file 1 — Supplementary Text Figs. S1 to S17 Legend for data S1 [file sciadv.adr8592_sm.pdf]

Science Advances
AAAS

Supplementary Materials for

Role of D-serine in intestinal ROS accumulation after sleep deprivation

Feng Zheng *et al.*

Corresponding author: Hao Qu, quhao@hfut.edu.cn; Lei Zheng, lzheng@hfut.edu.cn

Sci. Adv. **11**, eadr8592 (2025)
DOI: 10.1126/sciadv.adr8592

The PDF file includes:

Supplementary Text
Figs. S1 to S17
Legend for data S1

Other Supplementary Material for this manuscript includes the following:

Data S1

Figs. S1 to S17: extended technical descriptions of results

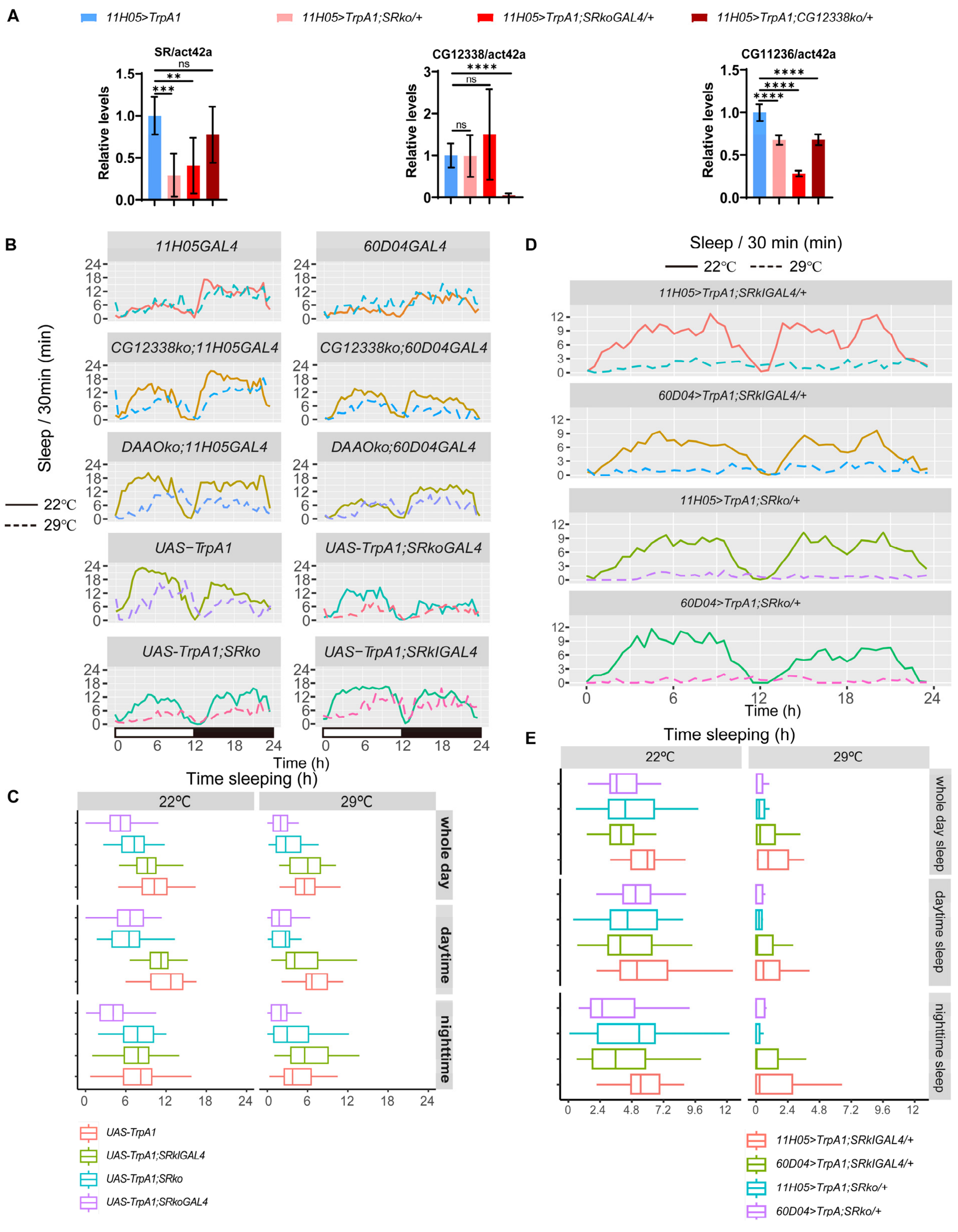

Fig. S1. The activation of SR-expressing cells does not lead to sleep deprivation. (A) Relative mRNA expression levels of SR, CG12338, and CG11236 in gene SR heterozygotes and gene

CG12338 heterozygote flies. (B) Sleep status of parental control flies at 22°C and 29°C, with consolidated sleep data from Day 1 and Day 2 at both temperatures. (C) Boxplot statistics for the sleep duration of UAS-TrpA1-associated flies at 22°C and 29°C, with significance labels omitted. (D) Sleep status of SRkIGAL4/+ and SRko/+ flies at 22°C and 29°C, with consolidated sleep data from Day 1 and Day 2 at both temperatures. (E) Boxplot statistics for sleep duration corresponding to (D), showing no significant differences between groups. Data are presented as means ± SEM or medians with the 25th and 75th percentiles. Statistical analyses were conducted using t-tests with Bonferroni-Dunn correction (A) and Pairwise Wilcoxon tests (B and D). ns indicates not significant; ** denotes $p < 0.01$; ***, $p < 0.001$; ****, $p < 0.0001$. Relevant statistical information is detailed in Data S1.

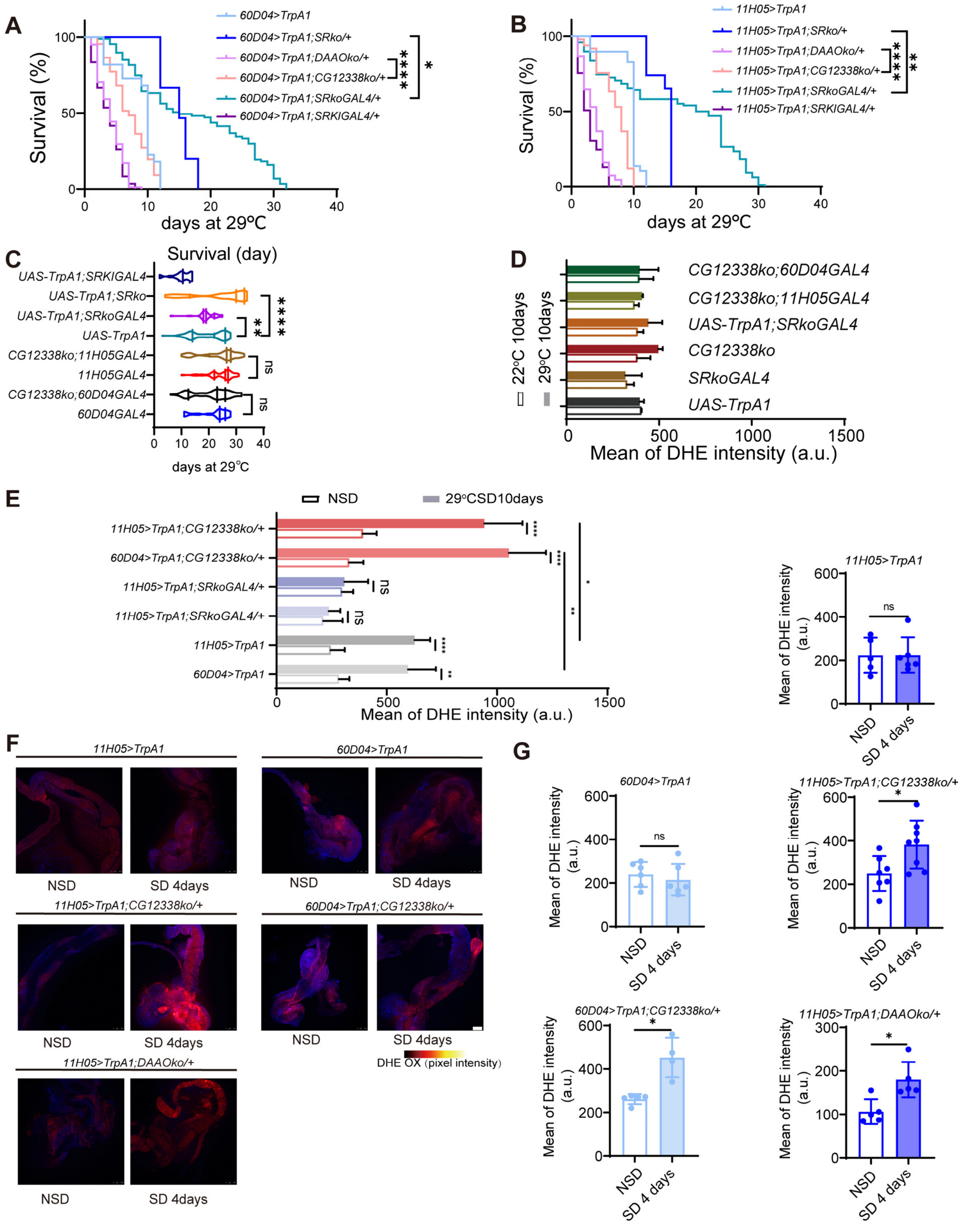

Fig. S2. Flies with SR deletion mutation do not exhibit increased gut ROS levels and show extended lifespan during sleep deprivation. (A-B) Survival analysis of thermogenetically sleep-

deprived flies indicates that SR knockdown prolongs lifespan, while DAAO knockdown shortens lifespan under sleep deprivation conditions. (C) Survival rates of parental line flies at 29°C. (D) Quantification of DHE intensity in parental lines at 22°C and 29°C. (E) Measurement of gut DHE intensity in flies subjected to thermogenetic sleep deprivation for 10 days, with CG12338ko/+ flies displaying increased ROS generation. (F) Representative confocal images of the gut showing oxidized DHE (DHE ox) in thermogenetically sleep-deprived flies on day 4, with scale bars set at 100 µm. The pseudo-color 'red hot' was applied for visualization. (G) Quantification of DHE intensity from the images in (F), revealing that DAAO knockdown in flies leads to the earlier onset of intestinal ROS during sleep deprivation. Data are presented as means ± SEM or medians with the 25th and 75th percentiles. Statistical analyses were conducted using log-rank tests (A to C), t-tests with Bonferroni-Dunn correction (D and E), ANOVA with Tukey's multiple comparisons test (E), and Mann-Whitney U tests (G). ns indicates not significant; ** denotes $p < 0.01$; ***, $p < 0.001$; ****, $p < 0.0001$. Relevant statistical information can be found in Data S1.

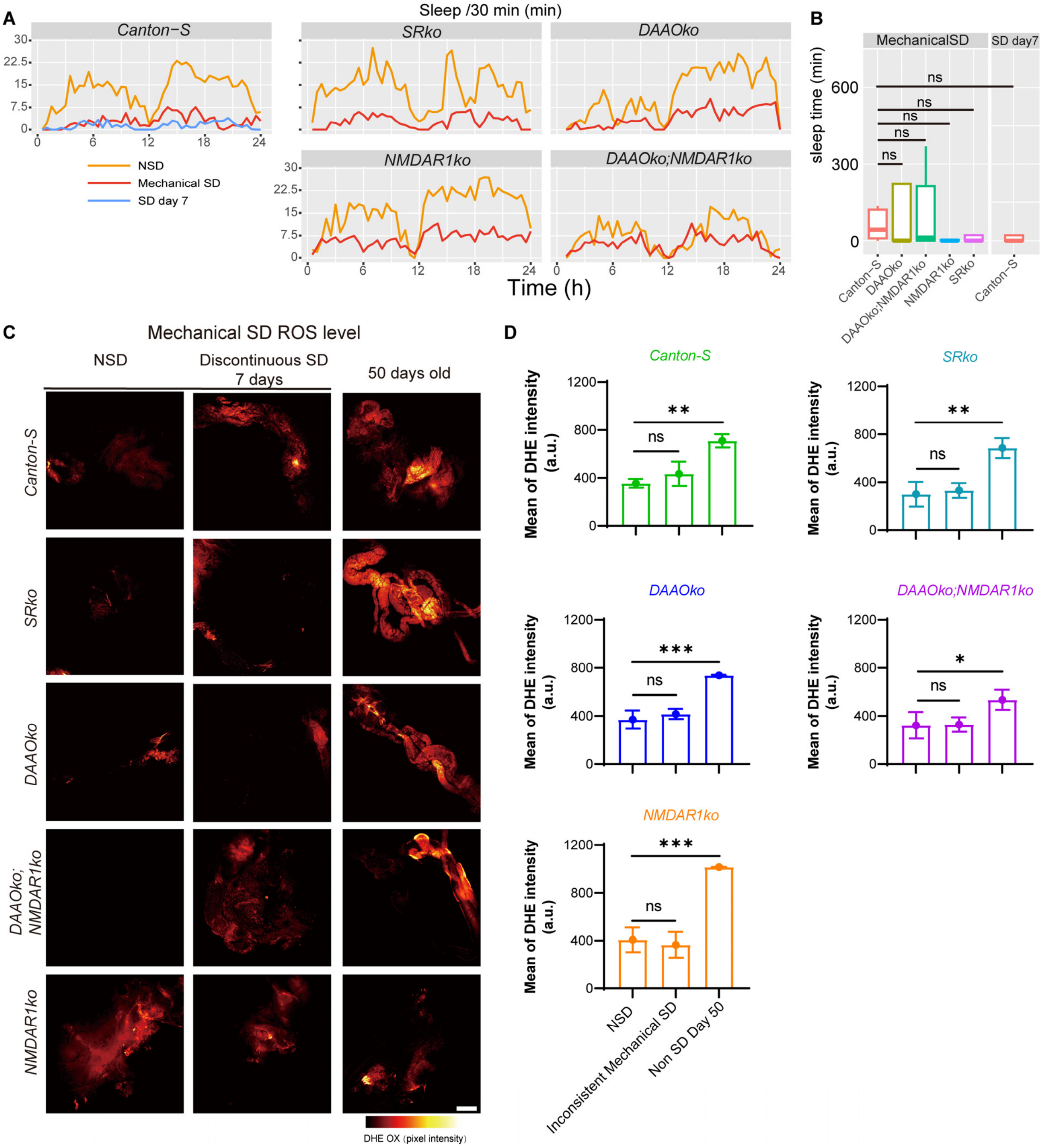

Fig. S3. Continuous mechanical SD is necessary for ROS accumulation. (A) Sleep status of flies subjected to mechanical vibration SD on Day 1 and Day 7. (B) Boxplot statistics for the sleep duration shown in (A), indicating that the effects of mechanical vibration SD were consistent over the 7-day period. (C) Representative confocal images of the gut displaying oxidized DHE (DHE ox) in flies subjected to inconsistent mechanical sleep deprivation for 7 days, compared to 50-day-old flies that had not experienced sleep deprivation. Scale bars represent 100 μm, and the pseudo-color 'glow' was applied for visualization. (D) Quantification of DHE intensity from the images in (C). Data are presented as means ± SEM or medians with the 25th and 75th percentiles. Statistical

analyses were conducted using Pairwise Wilcoxon tests (B) and ANOVA with Tukey's multiple comparisons test (D). ns indicates not significant; *, $p < 0.05$; **, $p < 0.01$; ***, $p < 0.001$. Relevant statistical information can be found in Data S1.

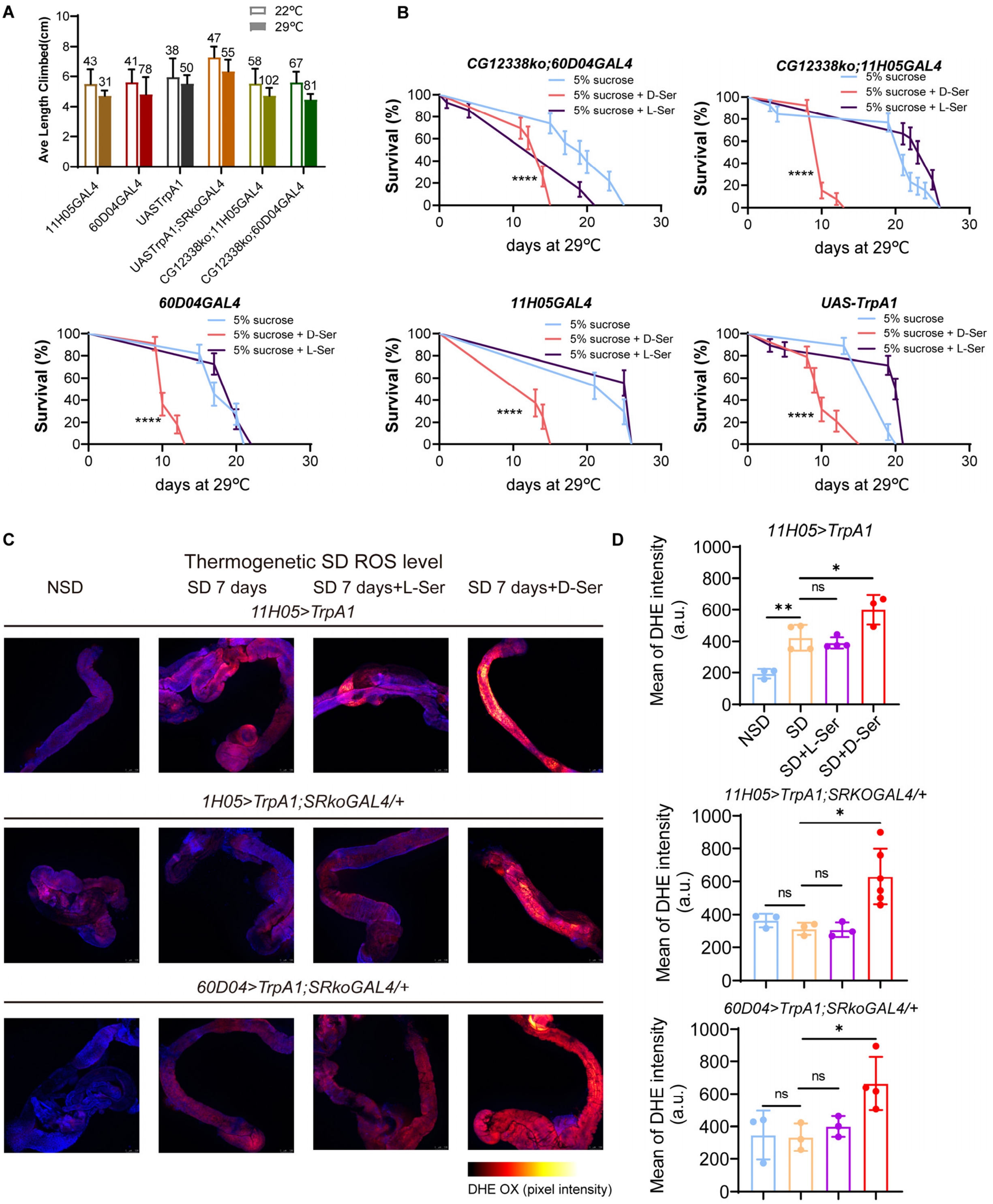

Fig. S4. Exogenous D-Ser supplementation accelerates gut ROS accumulation and increases mortality. (A) The Negative Geotaxis assay indicates that temperature does not impact the overall muscle or neuronal functions of the parental line flies. Sample sizes are indicated numerically. (B) Survival rates of parental line flies fed with 5% sucrose food additional L-Ser or D-Ser

supplements. (C) Representative confocal images of the gut showing oxidized DHE (DHE ox) in flies that received extra L-Ser or D-Ser supplementation after 7 days of sleep deprivation. Scale bars represent 100 μm, and the pseudo-color 'red hot' was applied for visualization. (D) Quantification of DHE intensity from the images in (C). Data are presented as means ± SEM. Statistical analyses were conducted using the Kruskal-Wallis test with Dunn's multiple comparisons test (A), log-rank test (B), and ANOVA with Tukey's multiple comparisons test (D). ns indicates not significant; *, $p < 0.05$; **, $p < 0.01$; ***, $p < 0.001$; ****, $p < 0.0001$. Relevant statistical information can be found in Data S1.

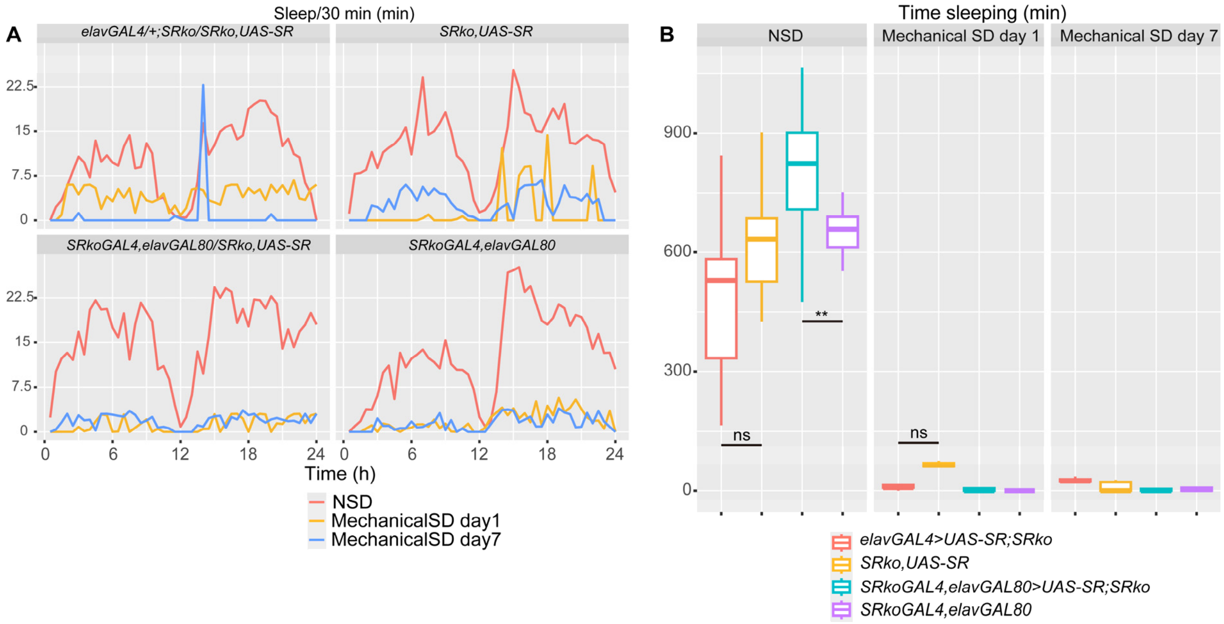

Fig. S5. The reintroduction of UAS-SR into various tissue within the SRkoGAL4 background does not influence the effects of SD. (A) Sleep status of flies subjected to mechanical vibration SD on Day 1 and Day 7. (B) Boxplot statistics for the sleep duration shown in (A). Data are presented as medians with the 25th and 75th percentiles. Statistical analysis was conducted using Pairwise Wilcoxon tests (B). ns indicates not significant; ** denotes $p < 0.01$. Relevant statistical information can be found in Data S1.

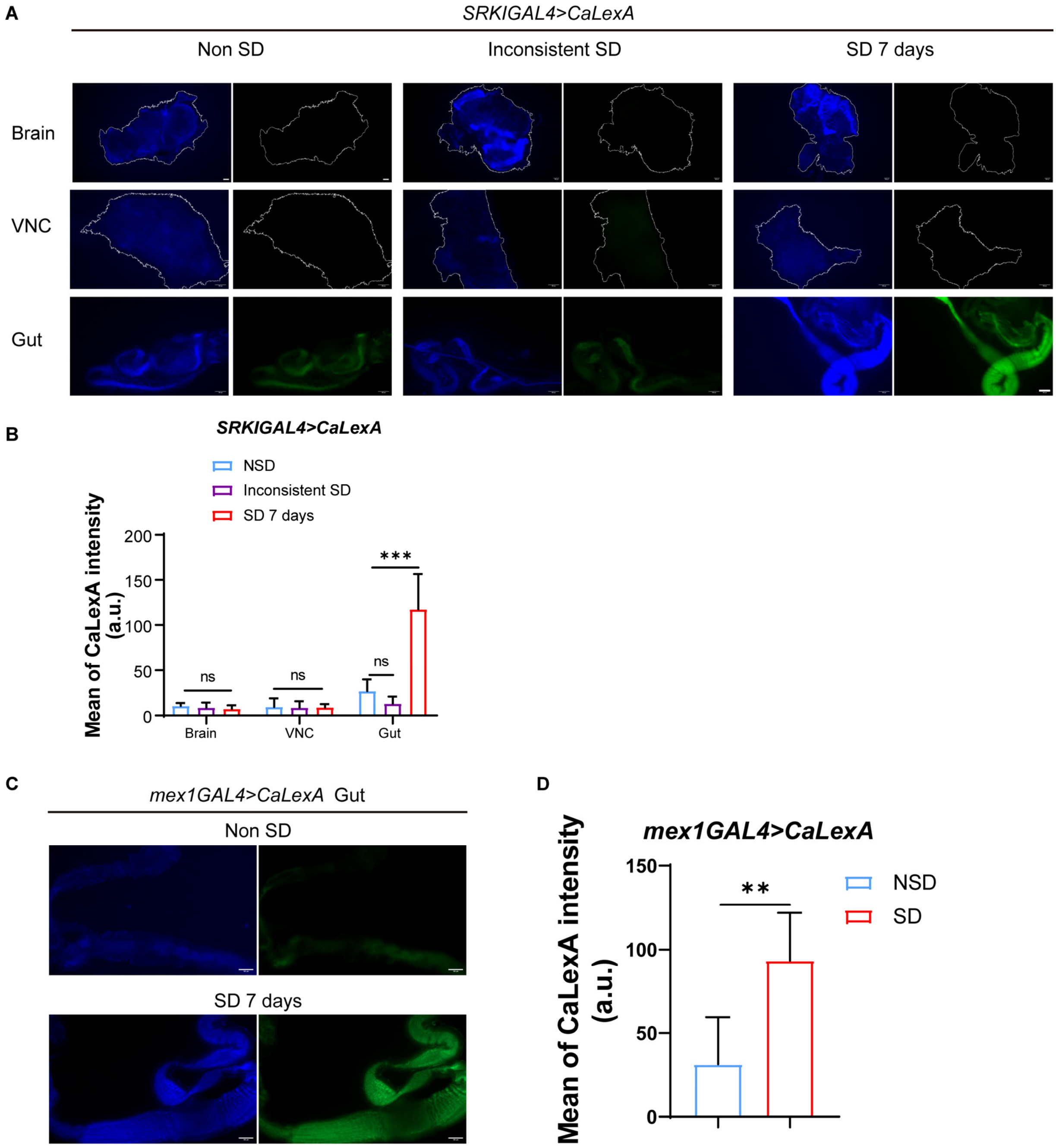

Fig. S6. Sleep deprivation enhances EC activity but does not affect brain or VNC activity. (A) Representative DAPI and GFP fluorescent images of the brain, VNC, and gut from sleep-deprived (SD), inconsistently sleep-deprived, and non-sleep-deprived (non-SD) flies. Scale bars represent 100 µm. (B) Quantification of GFP intensity from the images in (A). (C) Representative DAPI and GFP fluorescent images of the gut from SD and non-SD mex1GAL4>CaLexA flies. Scale bars represent 100 µm. (D) Quantification of GFP intensity from the images in (C). Data are presented as means ± SEM. Statistical analysis was conducted using t-tests with Bonferroni-Dunn

correction (B an D). ns indicates not significant; **, $p < 0.01$; ***, $p < 0.001$. Relevant statistical information can be found in Data S1.

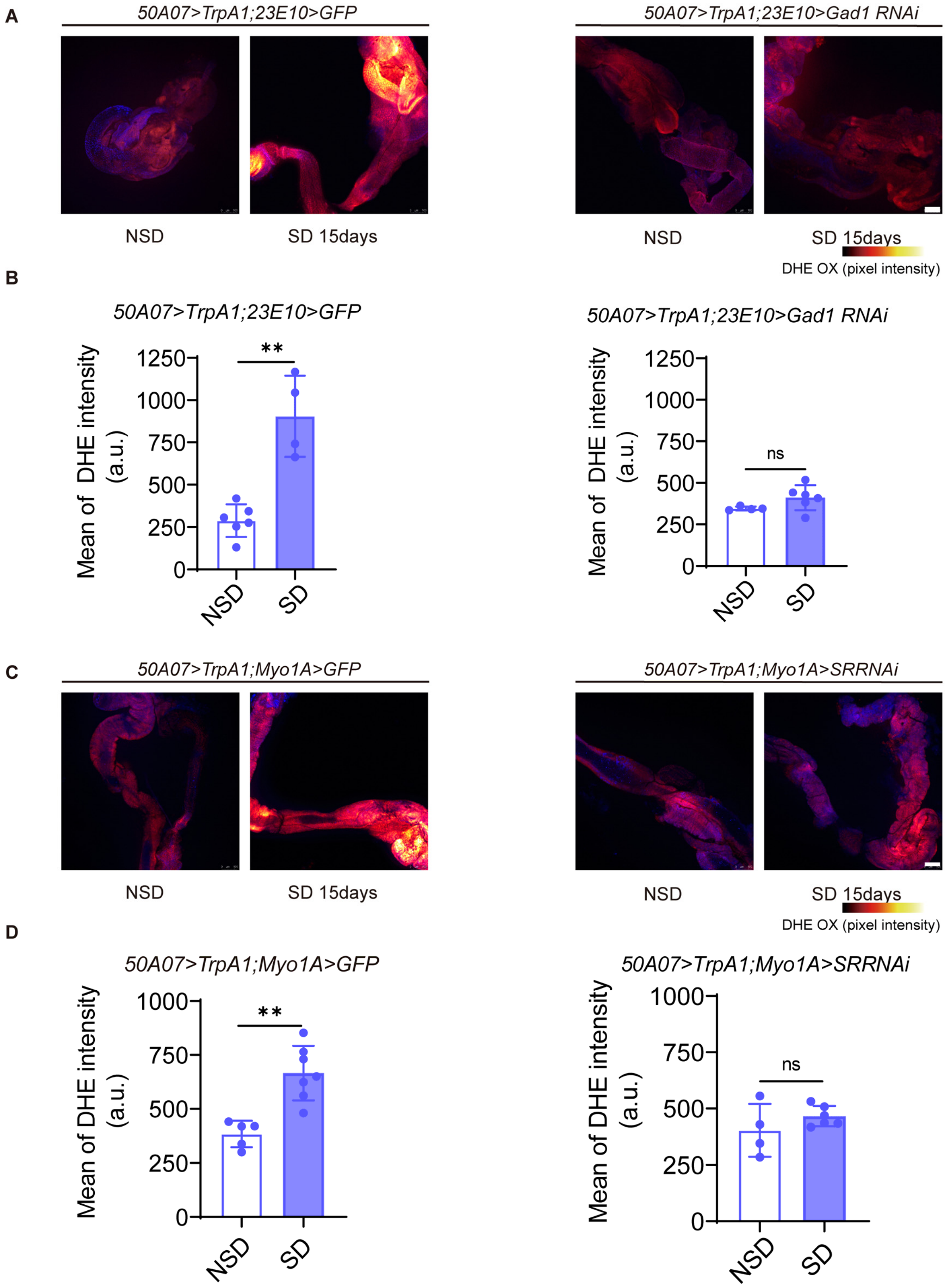

Fig. S7. RNAi-mediated knockdown of Gad1 in dFB neurons or SR in ECs does not lead to gut ROS accumulation caused by SD. (A) Representative confocal images of the gut showing oxidized DHE (DHE ox) from flies after 15 days of sleep deprivation. Scale bars represent 100

μm, and the pseudo-color 'red hot' was applied for visualization. (B) Quantification of DHE intensity from the images in (A). (C) Representative confocal images of the gut showing oxidized DHE (DHE ox) from flies after 15 days of sleep deprivation. Scale bars represent 100 μm, and the pseudo-color 'red hot' was applied for visualization. (D) Quantification of DHE intensity from the images in (C). Data are presented as means ± SEM. Statistical analysis was conducted using the Mann-Whitney U test (B an D). ns indicates not significant; ** denotes $p < 0.01$. Relevant statistical information can be found in Data S1.

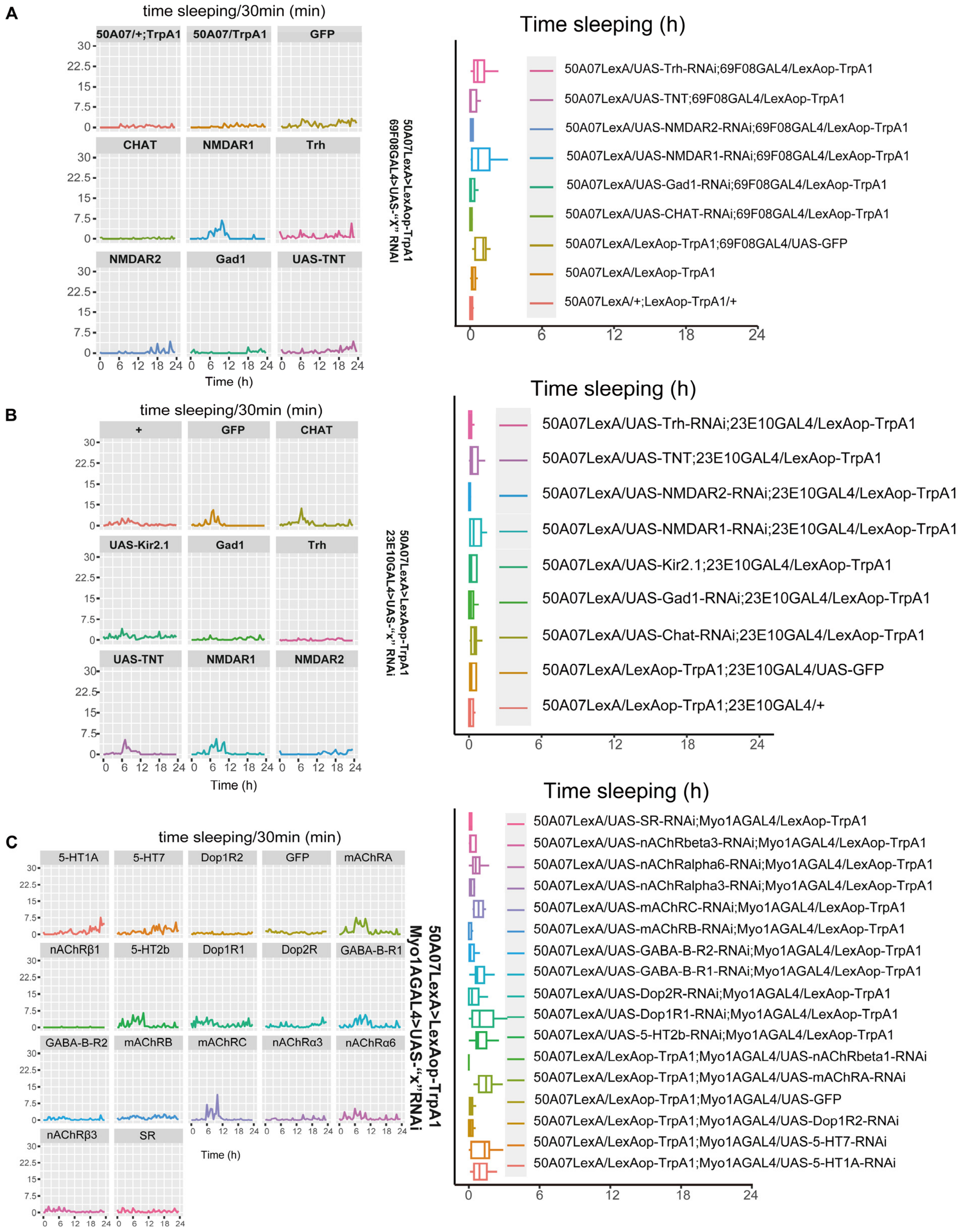

Fig. S8. Thermogenetic sleep data indicate that all screened lines exhibit SD. (A) Sleep status of lines assessed in the screening for R5 neurons at 29°C. (B) Sleep status of lines evaluated in the

screening for dFB neurons at 29°C. (C) Sleep status of lines analyzed in the screening for gut ECs at 29°C. Data are presented as medians with the 25th and 75th percentiles. Relevant statistical information can be found in Data S1.

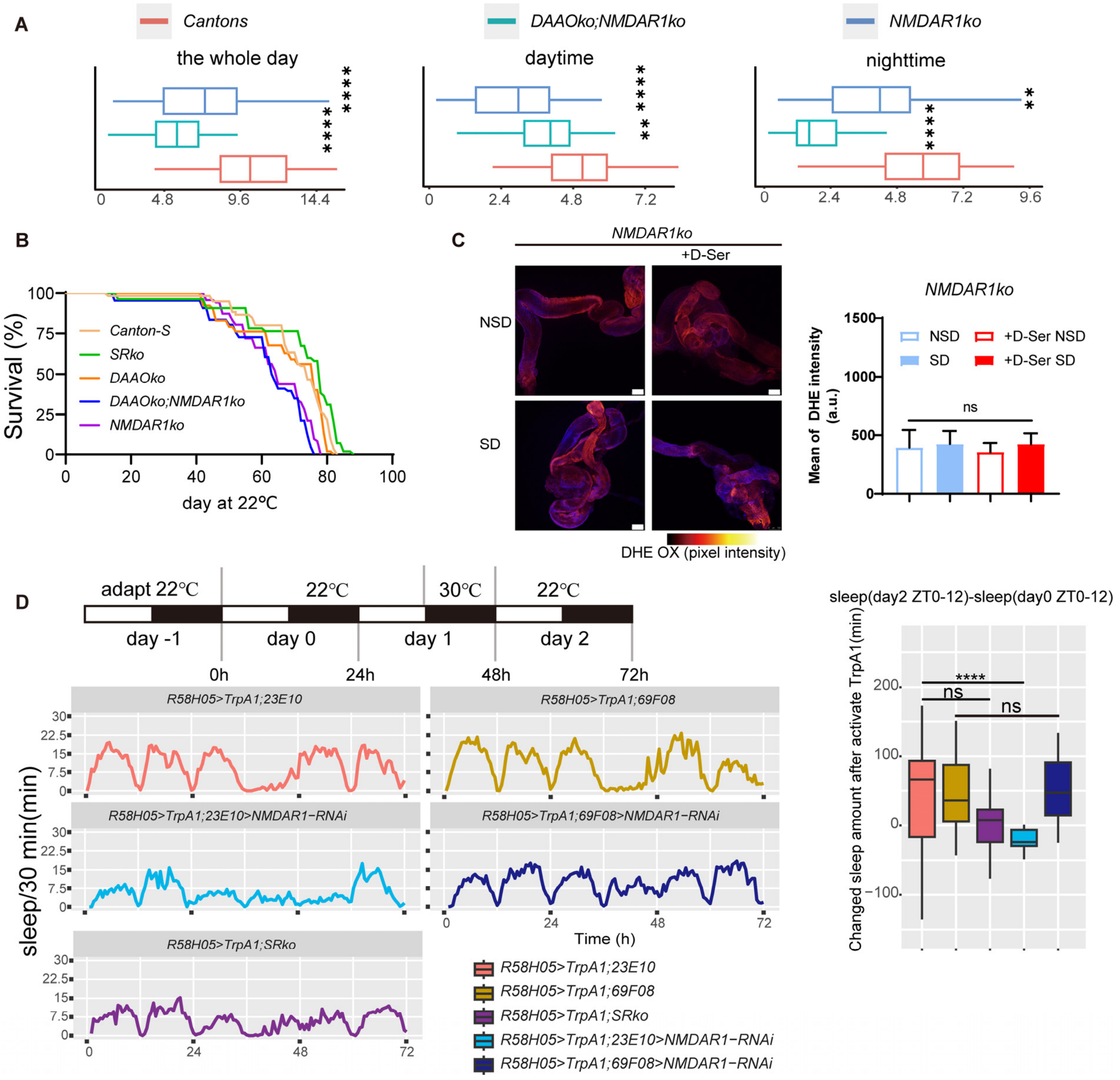

Fig. S9. Exogenous D-Ser supplementation with NMDAR1ko flies does not lead to gut ROS accumulation caused by SD. (A) Boxplot statistics for sleep duration of Canton-S, NMDAR1ko, and DAAOko;NMDAR1ko flies at 22°C, with sleep data consolidated over two days. The data for Canton-S is the same as shown in Figure 1. (B) Survival analysis of knockout lines for D-ser-related genes at 22°C. (C) Representative fluorescent images of gut DHE from SD and non-SD NMDAR1ko flies fed with food contain D-Ser or with normal food. DHE intensity had been quantified. Scale bars represent 100 μm, with pseudo-color "red hot" applied. (D) Rebound sleep following stimulation of the R58H05LexA driver requires NMDAR1 in dFB neurons. Genotypes: R58H05LexA/+;LexAop-TrpA1/23E10GAL4, R58H05LexA/+;LexAop-TrpA1/69F08GAL4, R58H05LexA/LexAop-TrpA1;SRko/SRko, R58H05LexA/UAS-NMDAR1-RNAi;LexAop-TrpA1/23E10GAL4, R58H05LexA/UAS-NMDAR1-RNAi;LexAop-TrpA1/69F08GAL4. Data are presented as means ± SEM or as medians with the 25th and 75th percentiles. Statistical analyses were performed using the Pairwise Wilcoxon test (A and D), the ANOVA test with tukey's

multiple comparisons test (C) and log-rank tests (B). ns indicates not significant; *, $p < 0.05$; **, $p < 0.01$; ****, $p < 0.0001$. Relevant statistical information can be found in Data S1.

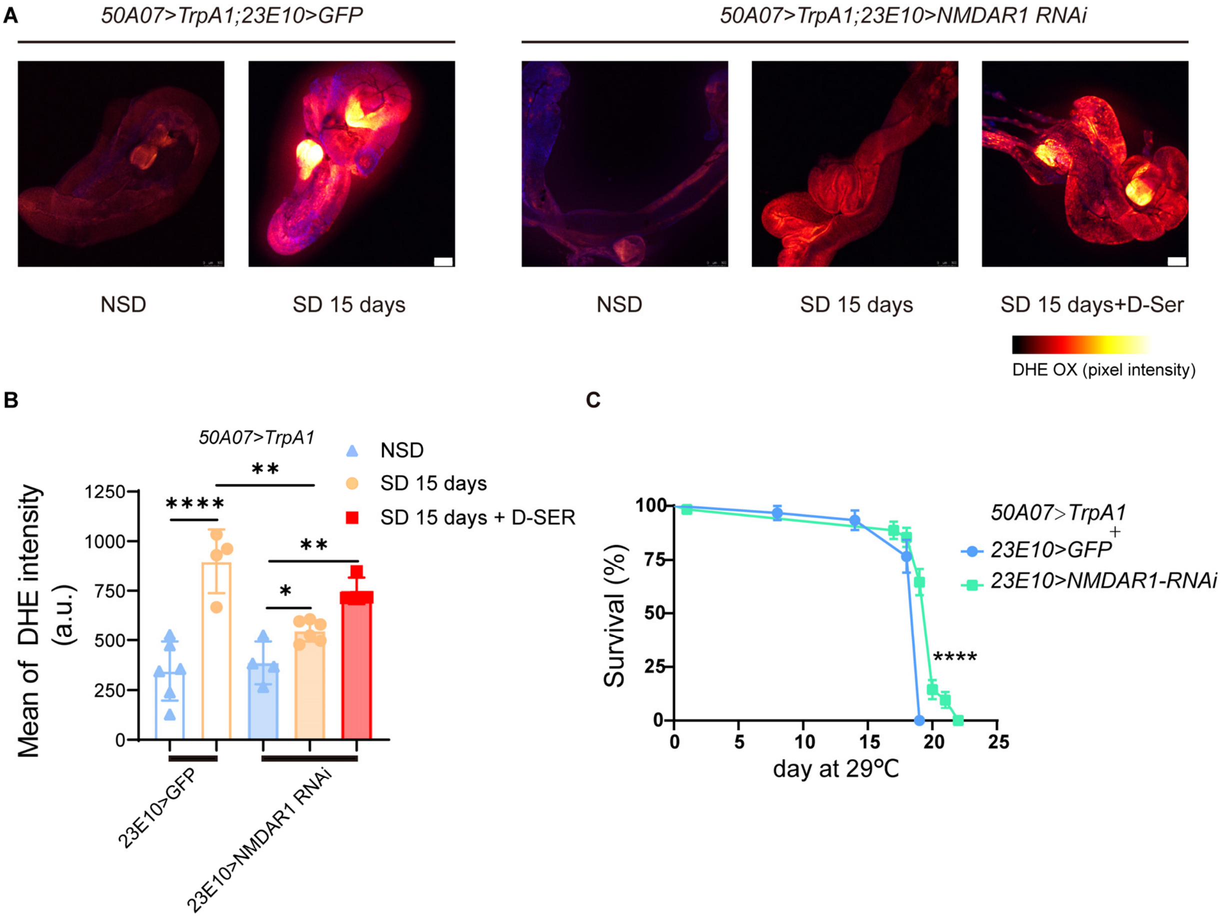

Fig. S10. RNAi-mediated knockdown of NMDAR1 in dFB neurons reduces gut ROS accumulation and slows down the rate of death caused by SD. (A) Representative confocal images of the gut showing oxidized DHE (DHE ox) in flies fed with food contain D-Ser or with normal food after 15 days of sleep deprivation. Scale bars represent 100 μm, and the pseudo-color 'red hot' was applied for visualization. (B) Quantification of DHE intensity from the images in (A). (C) Survival data indicate that NMDAR1 RNAi in dFB neurons extends the lifespan of flies under SD conditions. Data are presented as means ± SEM. Statistical analyses were performed using the ANOVA test with tukey's multiple comparisons test (B) and log-rank tests (C). ns indicates not significant; *, $p < 0.05$; **, $p < 0.01$; ****, $p < 0.0001$. Relevant statistical information can be found in Data S1.

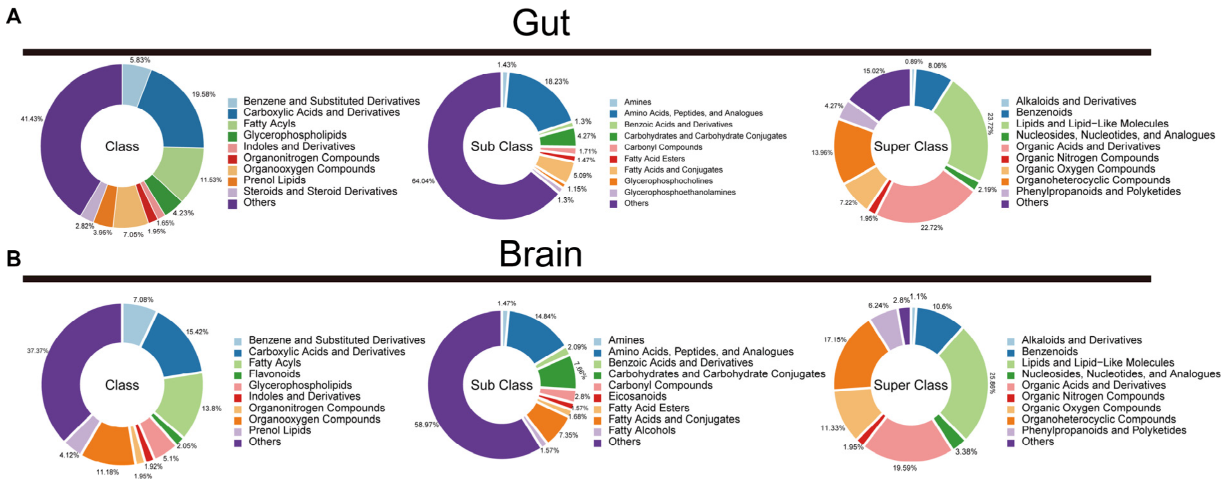

Fig. S11. Statistics of metabolomics data. (A) Overview of the identified metabolite classes in gut samples. (B) Overview of the identified metabolite classes in brain samples.

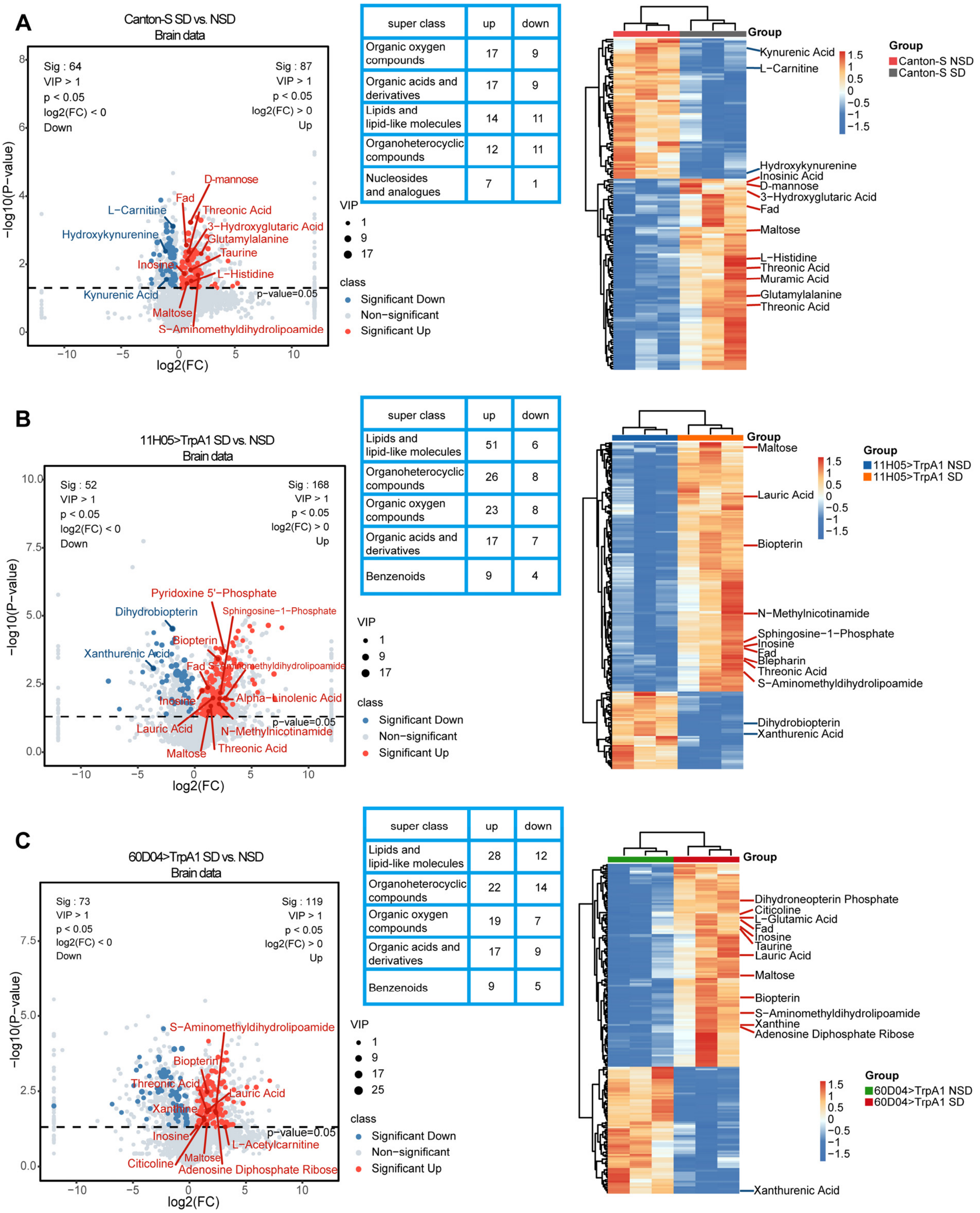

super class	up	down
Organic oxygen compounds	17	9
Organic acids and derivatives	17	9
Lipids and lipid-like molecules	14	11
Organoheterocyclic compounds	12	11
Nucleosides and analogues	7	1

super class	up	down
Lipids and lipid-like molecules	51	6
Organoheterocyclic compounds	26	8
Organic oxygen compounds	23	8
Organic acids and derivatives	17	7
Benzenoids	9	4

super class	up	down
Lipids and lipid-like molecules	28	12
Organoheterocyclic compounds	22	14
Organic oxygen compounds	19	7
Organic acids and derivatives	17	9
Benzenoids	9	5

Fig. S12. The impact of sleep deprivation on the brain metabolome of the flies. (A) Volcano and heatmap showing changes in expression of brain metabolites hit in *Canton-S* (SD vs. NSD)group data. (B) Volcano and heatmap showing changes in expression of brain metabolites

hit in *11H05>TrpA1* (SD vs. NSD)group data. (C) Volcano and heatmap showing changes in expression of brain metabolites hit in *60D04>TrpA1* (SD vs. NSD)group data.

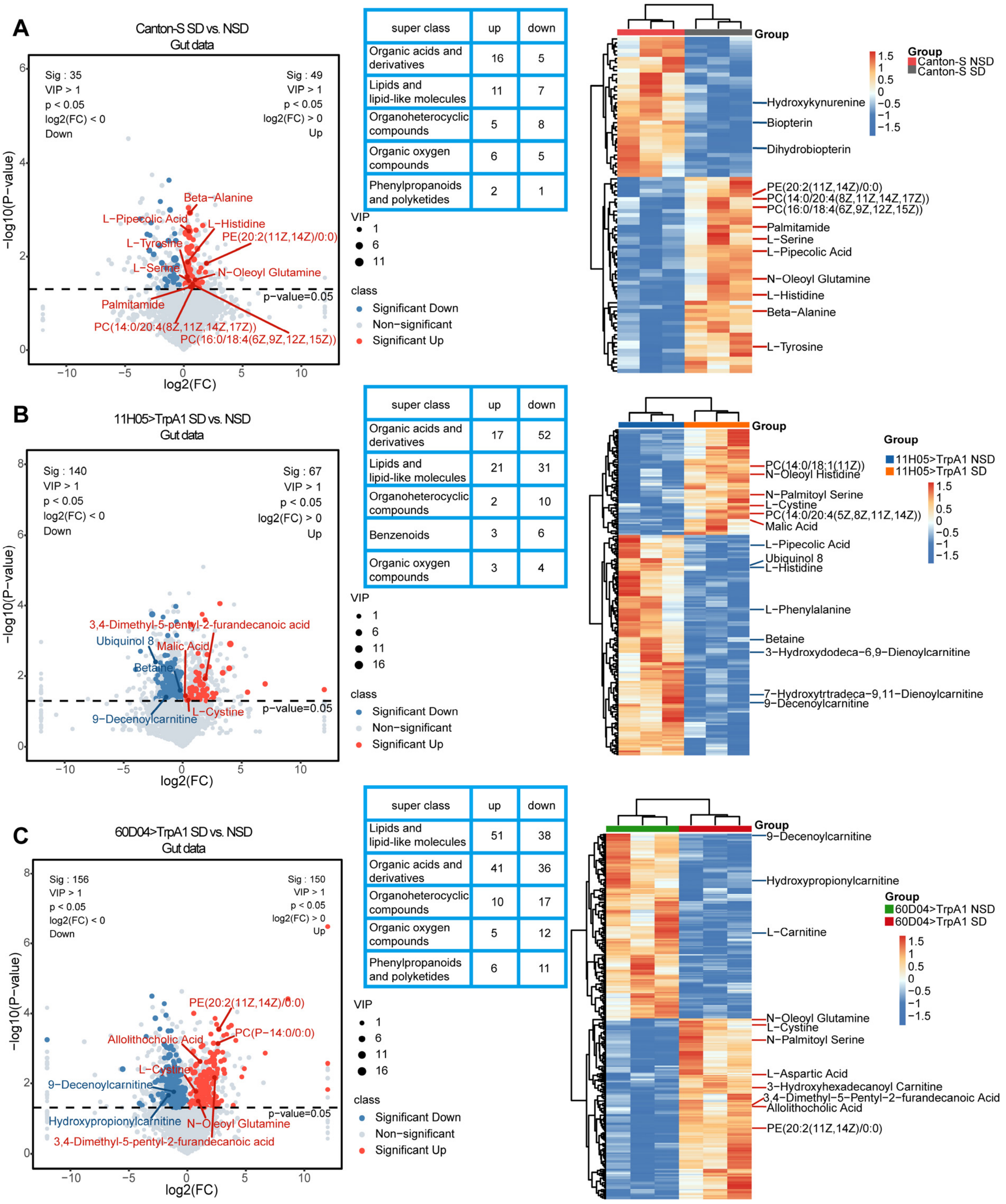

Fig. S13. The impact of sleep deprivation on the gut metabolome of the flies. (A) Volcano and heatmap showing changes in expression of gut metabolites hit in *Canton-S* (SD vs. NSD)group data. (B) Volcano and heatmap showing changes in expression of gut metabolites hit in

11H05>TrpA1 (SD vs. NSD)group data. (C) Volcano and heatmap showing changes in expression of gut metabolites hit in *60D04>TrpA1* (SD vs. NSD)group data.

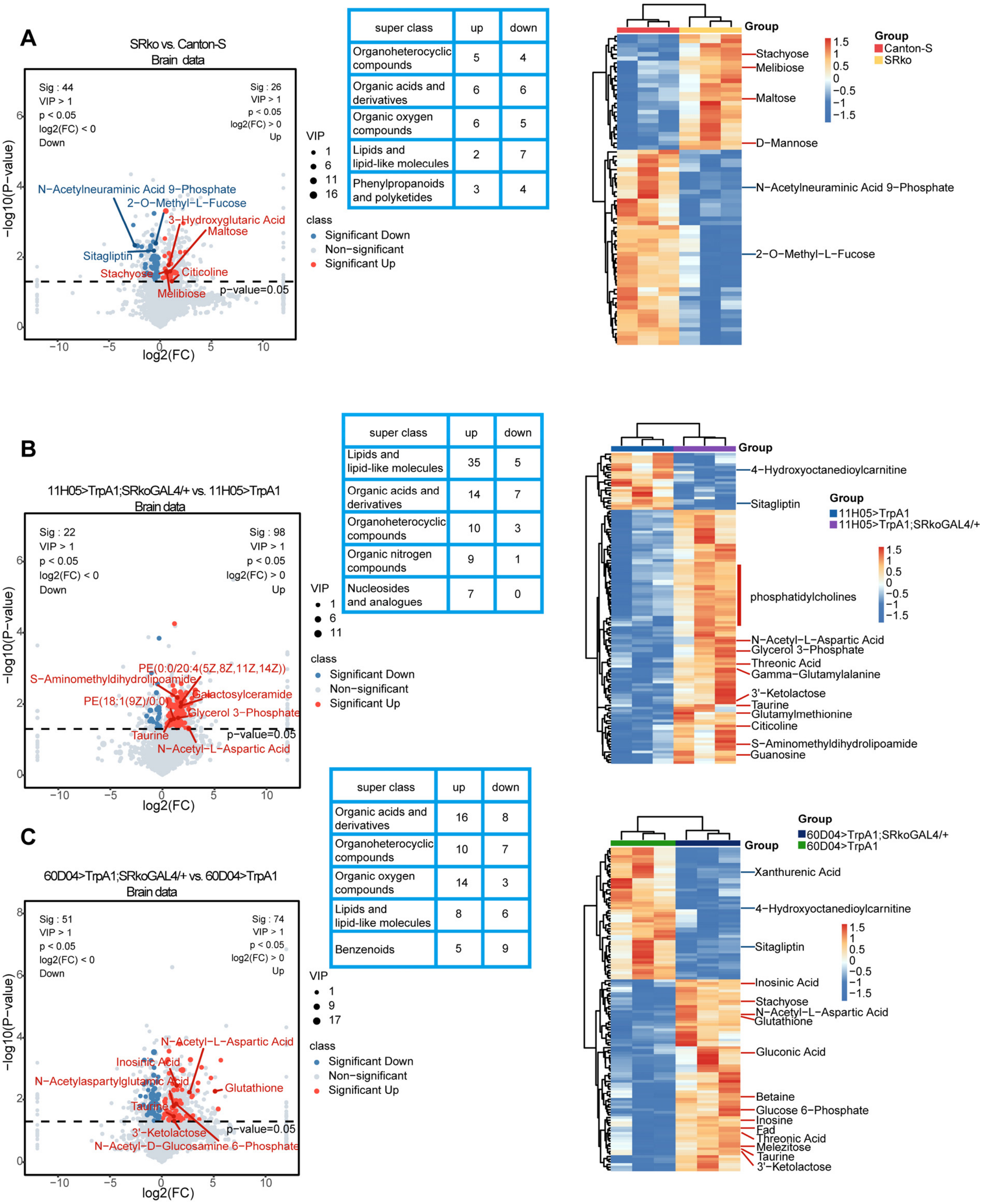

super class	up	down
Organoheterocyclic compounds	5	4
Organic acids and derivatives	6	6
Organic oxygen compounds	6	5
Lipids and lipid-like molecules	2	7
Phenylpropanoids and polyketides	3	4

super class	up	down
Lipids and lipid-like molecules	35	5
Organic acids and derivatives	14	7
Organoheterocyclic compounds	10	3
Organic nitrogen compounds	9	1
Nucleosides and analogues	7	0

super class	up	down
Organic acids and derivatives	16	8
Organoheterocyclic compounds	10	7
Organic oxygen compounds	14	3
Lipids and lipid-like molecules	8	6
Benzenoids	5	9

Fig. S14. The impact of SR knockout on the brain metabolome of the flies. (A) Volcano and heatmap showing changes in expression of brain metabolites hit in *SR* vs. *Canton-S* group data. (B) Volcano and heatmap showing changes in expression of brain metabolites hit in *11H05>TrpA1;SRkoGAL4/+* vs. *11H05>TrpA1* group data. (C) Volcano and heatmap showing

changes in expression of brain metabolites hit in *60D04>TrpA1;SRkoGAL4/*+ vs. *60D04>TrpA1* group data.

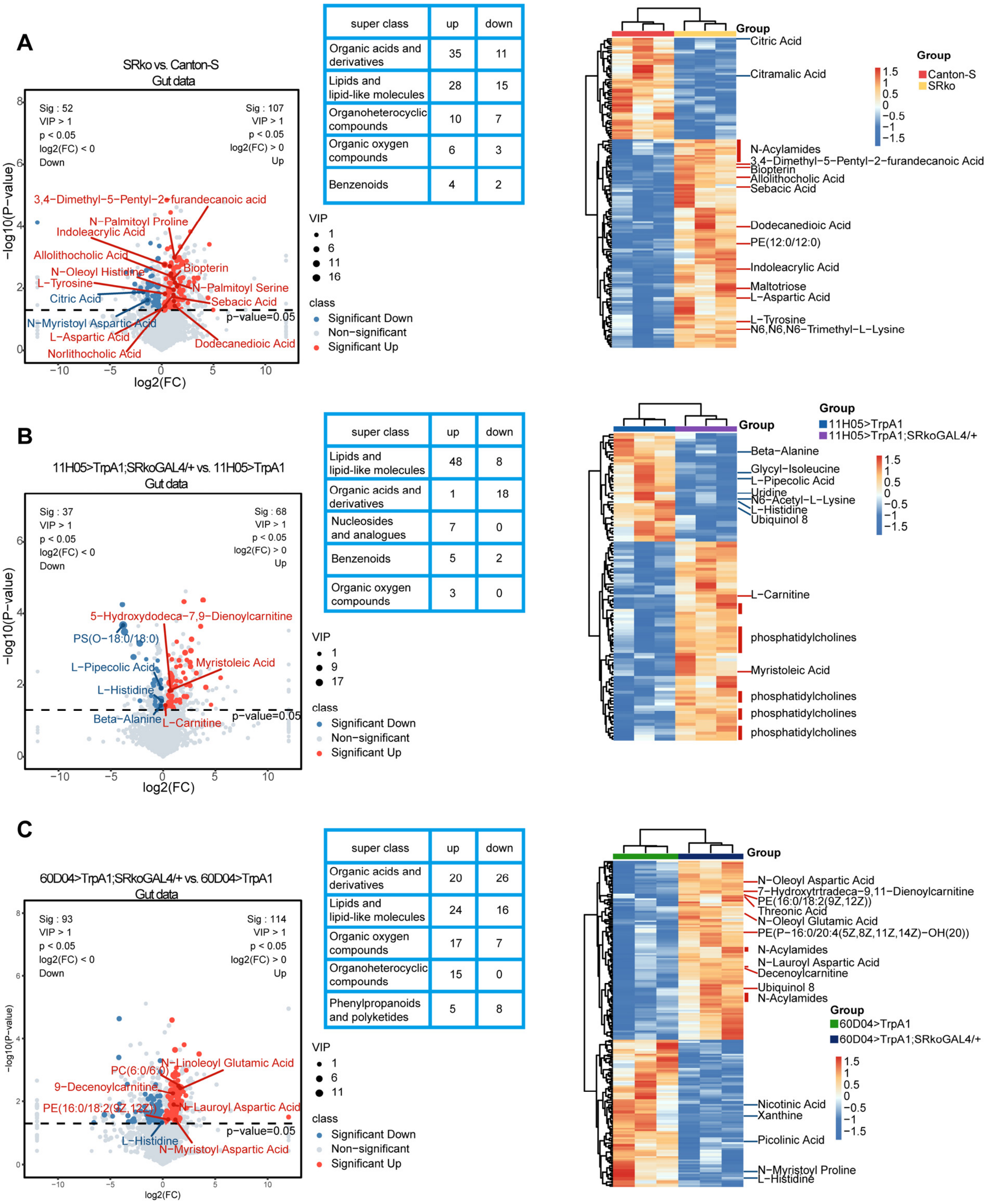

Fig. S15. The impact of SR knockout on the gut metabolome of the flies. (A) Volcano and heatmap showing changes in expression of gut metabolites hit in *SR* vs. *Canton-S* group data. (B) Volcano and heatmap showing changes in expression of gut metabolites hit in *11H05>TrpA1;SRkoGAL4/+* vs. *11H05>TrpA1* group data. (C) Volcano and heatmap showing

changes in expression of gut metabolites hit in *60D04>TrpA1;SRkoGAL4/+* vs. *60D04>TrpA1* group data.

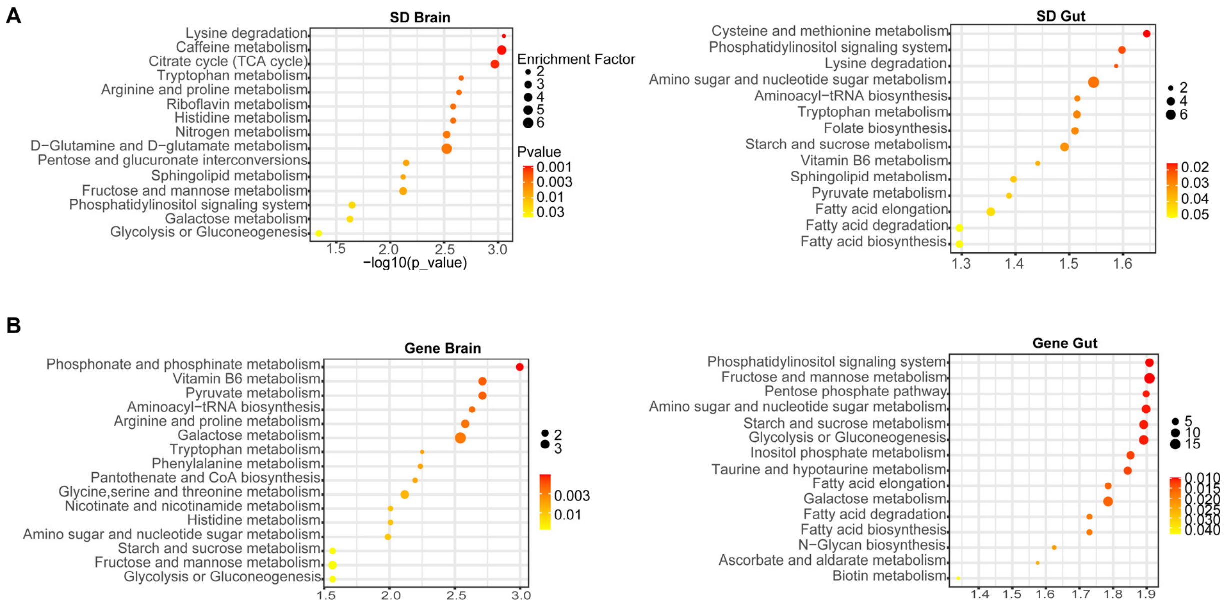

Fig. S16. Sleep deprivation or SR knockout affect the fatty acids metabolism in the gut of flies, but not in its brain. (A) Results from the enrichment analysis of a single factor (sleep deprivation) in both gut and brain samples. (B) Results from the enrichment analysis of a single factor (SR knockout) in both gut and brain samples.

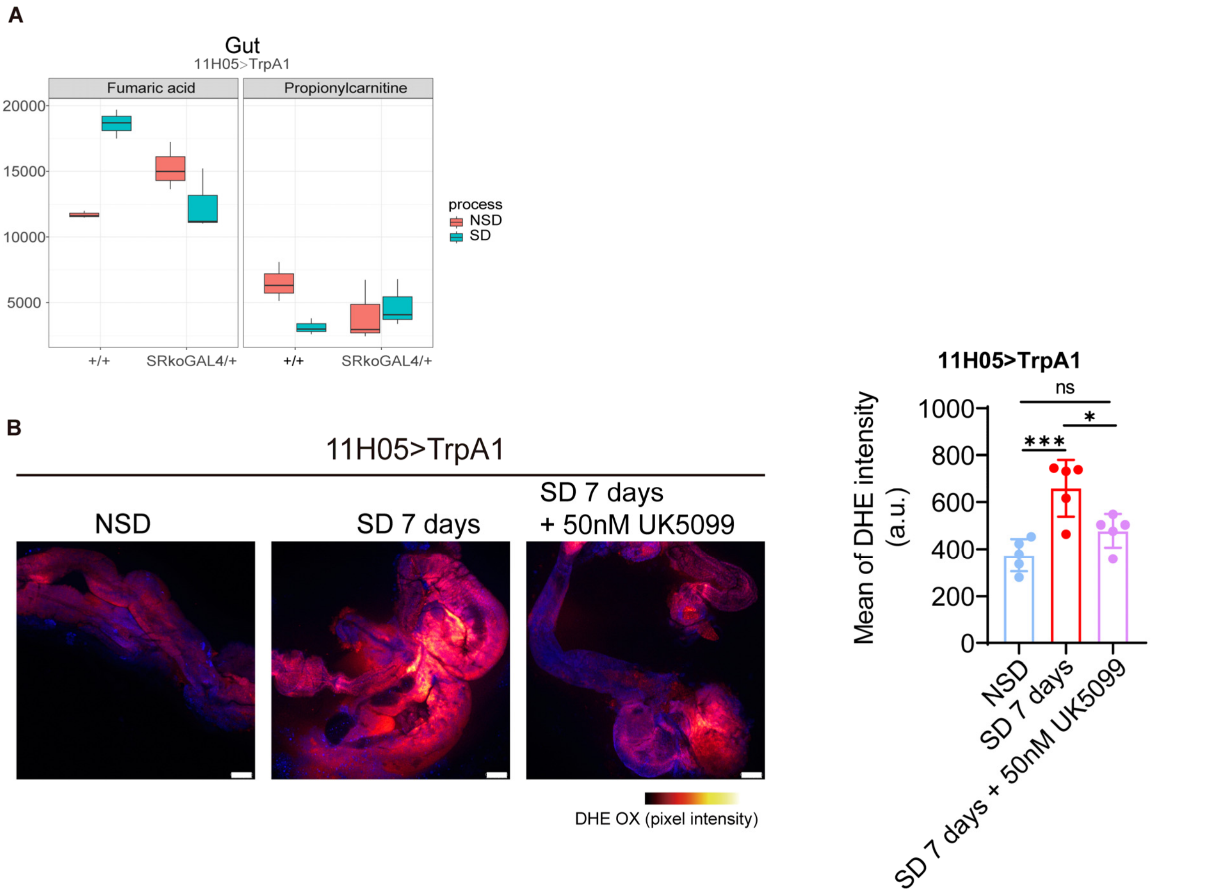

Fig. S17. Inhibition of pyruvate entry into mitochondria reduces gut ROS accumulation caused by SD. (A) Changes in fumaric acid and propionylcarnitine levels across different subgroups. (B) Representative confocal images of the gut showing oxidized DHE (DHE ox) from flies fed supplemental UK5099 after 7 days of SD and quantification of DHE intensity from the images. Scale bars represent 100 μm, with the pseudo-color 'red hot' applied for visualization. Data are presented as means ± SEM. Statistical analysis was conducted using ANOVA with Tukey's multiple comparisons test (B). ns indicates not significant; *, $p < 0.05$; ***, $p < 0.001$. Relevant statistical information can be found in Data S1.

Data S1. (separate file)

Supplementary tables contain notes clarifying statistical analyses
